# Rapid and efficient labeling by a selective organic fluorophore probe highlights heterogeneity of mycobacterial populations and persister resuscitation

**DOI:** 10.1371/journal.pone.0338563

**Published:** 2025-12-18

**Authors:** Priyanka Chauhan, Sander van Otterdijk, Susanna Commandeur, Dirk Bald, Frank J. Bruggeman

**Affiliations:** Amsterdam Institute for Life and Environment (A-LIFE), AIMMS, Faculty of Science, Vrije Universiteit Amsterdam, HZ Amsterdam, The Netherlands; Rutgers Biomedical and Health Sciences, UNITED STATES OF AMERICA

## Abstract

Stress tolerant slow- or non-growing bacterial subpopulations represent a key factor for chronic, recurrent, antibiotic-tolerant infections and necessitate prolonged antibiotic therapy. Consequently, new tools that facilitate investigation of these small subpopulations are highly needed. To characterize slow-/non-growing populations of mycobacteria, we here utilized Vybrant DiD, a lipophilic, fluorescent organic probe that detected pathogenic and non-pathogenic species in a rapid, specific and non-invasive manner. It enabled accurate, direct quantification of mycobacterial replication rate *in vitro* and in murine macrophages at the population and single-cell level, providing insight into population heterogeneity. We determined the preexisting slow-/non-growing fraction of mycobacteria during exponential growth, which increased upon stress. Monitoring mycobacterial resuscitation after antibacterial treatment and after low-oxygen-induced dormancy revealed the stochastic and heterogeneous nature of the resuscitation process. We anticipate that this method will be widely utilized in basic research on bacterial persistence and may also be included in applied settings, e.g., high-throughput drug characterization.

## Introduction

Phenotypic heterogeneity, defined as physiologically distinct subpopulations within an isogenic population, occurs in many bacterial species [1]. It enhances the fitness of a bacterial population by bet-hedging, allowing for subpopulation survival in dynamic and stressful conditions [2]. The phenomenon of phenotypic heterogeneity, in particular the existence of stress-tolerant slow-/non-growing bacterial subpopulations, is all-the-more relevant for pathogenic bacteria as a strategy to adapt, survive and persist under diverse physiological and immunological microenvironments within the host during infection [3]. One such important class of pathogenic bacteria are mycobacteria, which include *Mycobacterium tuberculosis*, the causative agent of tuberculosis, *Mycobacterium ulcerans*, which causes buruli ulcer, *Mycobacterium avium*, an opportunistic pathogen associated with cystic fibrosis, and *Mycobacterium marinum,* causing tuberculosis-like infections in fish and skin lesions in humans. Mycobacteria can establish a long-term infection in the host that is associated with phenotypically diverse and drug-tolerant subpopulations during pathogenesis [4]. These surviving subpopulations of cells are a major reason for chronicity of infections that could beget resistance, for relapse of disease and for the prolonged antibiotic therapy [5,6]. For instance, tuberculosis treatment requires at least 4 months [7].

Hence, quantification and characterization of slow-/non-growing subpopulation is important for development of improved and shortened treatment strategies and the design of new antimicrobials. Nevertheless, since these subpopulations can vary in size by orders of magnitude, their quantification poses a significant challenge. To address this issue, development of rapid and highly sensitive methods suitable for high-throughput applications under therapeutically relevant conditions are needed.

Recent advancements in single-cell technologies and the growing arsenal of genetically-engineered fluorescent reporter plasmids and strains have enabled studies of phenotypic heterogeneity in the field of mycobacteriology [8,9]. Using microfluidics in conjugation with time-lapse microscopy and a 16S rRNA based reporter, Manina et al. observed heterogeneity of growth rate and gene expression at single-cell level in *M. tuberculosis* grown under standard growth conditions, which was further amplified by stress conditions and murine infection [10]. In another report, a dual replication reporter system based on fluorescence dilution was utilized to study replication rate and population heterogeneity of mycobacteria under various growth conditions [11]. Alternatively, a dual-reporter mycobacteriophage system was utilized to characterize heterogeneity of gene expression among an INH-tolerant population in laboratory and clinical *M. tuberculosis* cultures [12]. Recently, a promising method called PerSort was developed to isolate and characterize subpopulations of *Mycobacterium smegmatis* dormant persister cells that preexist in small numbers with *Mycobacterium* cultures. [13]. This fluorescence reporter plasmid-based method utilizes low translational activity of persister cells relative to actively growing cells to sort the subpopulations using flow cytometry [13].

Although reporter- or plasmid-based methods have been useful for visualizing and analyzing multiple aspects of bacterial physiology and virulence [9], their development is hampered by slow growth and the pathogenicity of some mycobacterial species [14,15]. Moreover, fluorescent proteins cannot label molecular species not directly encoded by genes, like peptidoglycans and lipid bilayers [14]. Hence, labeling methods based on fluorescent organic dyes hold promise as alternative tools. While large arrays of probes have been developed for use in mammalian cells [16–19], only relatively few have been developed for bacteria [14].

Here, we developed and validated a labeling approach to study mycobacterial growth dynamics and phenotypic heterogeneity using a fluorescent organic probe, that up to now has mainly been used to study mammalian cells. Vybrant DiD (Molecular Probes, USA) is a highly lipophilic probe that exhibits fluorescence upon insertion into a lipid environment. This probe originally was developed to circumvent obstacles of uniform cellular labeling in aqueous culture media, requirement of specific buffer for labeling and reversible diffusion that leads to secondary labeling of nearby cells. We show that assays developed on this probe are robust and compatible with both flow cytometry and fluorescence microscopy. This approach can selectively label live pathogenic and non-pathogenic mycobacterial species within 5–20 min using a minimally invasive protocol. The high sensitivity (far-red rather than green emitting) and stability of Vybrant DiD, with minimal detachment from targeted cells and no effect on bacterial viability, can make this approach valuable for rapid mycobacterial detection and visualization. This labeling approach was successfully utilized to assess growth and division in various *in vitro* and *ex vivo* growth conditions at population and single-cell level, allowing for robust quantification of preexisting slow-/non-growing subpopulations. The Vybrant DiD was also utilized to assess the relation between stress duration and resuscitation dynamics, providing insight into these important parameters in mycobacterial biology.

## Materials and methods

### Bacterial strains, media and culturing conditions

*Mycobacterium smegmatis mc*^*2*^ *155*, *M. smegmatis* expressing DsRed (Msm-DsRed, [20]) and *Mycobacterium marinum* was cultured in Middlebrook 7H9 medium (Difco) containing 0.05% Tween-80 supplemented with 1 0% (vol/vol) Middlebrook ADC (Albumin-dextrose-catalase, BD Difco^TM^) Enrichment (7H9-ADC) at 37 °C and 30 °C, respectively. To culture *Mycobacterium tuberculosis* 6020 (Mtb), 7H9 medium supplemented with 10% OADC enrichment (oleic acid-albumin-dextrose-catalase, BD Difco^TM^), 0.02% Tyloxapol, 0.2% Casaminoacids, 0.24 mg/ml Pantothenate and 0.8 mg/ml L-lysine was used at 37°C. *Corynebacterium glutamicum* (Cg) cells were grown in complex medium consisting of Brain-Heart-Infusion (BD Difco^TM^ BHI) at 30 °C. To culture, *Bacillus subtilis* (Bs), *Pseudomonas aeruginosa* (Pa), *Escherichia coli* (Ec) and *Staphylococcus aureus* (Sa) were grown in LB media at 37 °C. All bacterial strains were grown under shaking conditions.

For each experiment, a standard culturing procedure was used: pre-culture from single colonies was first fully grown, the culture was then diluted to 1:1000 in fresh media and grown till exponential phase (~0.4 OD_600_). The primary culture was diluted to 1:100 in fresh media, grown till exponential phase (~0.4 OD_600_) and used for the experiment. Hence, the culture was maintained in exponential phase for at least 2 cycle of culturing before using it for experiment to minimize the effect of carryover of persister cells from stationary phase culture.

For growth curve analysis of *M. smegmatis* in liquid medium, The culture is diluted to 0.01 OD_600_ and growth was monitored by measuring OD_600_ at various time points. For plating *M. smegmatis* cells on agar, 7H9 agar plates supplemented with 10% (vol/vol) ADC enrichment (Albumin-Dextrose-Catalase, Difco) and 0.4% activated charcoal were used.

To expose the *M. smegmatis* cells to the low oxygen environment, Wayne model was utilized as described earlier with a few modifications [21]. Briefly, culturing was carried out in 120 mL serum bottles using 7H9-ADC media at 37 °C. Eighty ml of culture was used throughout and, therefore, the ratio of head space air volume (40 ml) to liquid volume (80 ml) was always 0.5. Rubber stoppers and aluminum crimp seal (without septum) were used to seal the bottles which permitted use of injection for taking out the sample or addition of reagents when required. Aerated control was cultured in similar manner except was not sealed rather cotton plugged to allow unlimited supply of oxygen and ¾ head space was maintained. The cultures was stirred gently using magnetic stirring bars at 170 rpm in order to allow slow diffusion of oxygen from the head space into the liquid culture. Along with qualitative resazurin anaerobic indicator strip, gas chromatography was used to monitor the oxygen level in the culture. Briefly, to measure O_2_ concentration in the headspace of the serum bottles a Shimadzu Tracera GC-2010 Plus chromatograph fitted with a Carboxen 1010 (30 m × 0.58 mm × 30 µm) column with helium as carrier gas and a barrier ionization detector (BID) was used. The injection size of each sample was 10 µL. The injector temperature was set at 250 °C to assure fast evaporation of the samples. The temperature of the column was initially programmed at 105 °C for a holding time of 7 min. After this, the temperature was incremented to 200 °C with a rate of 100 °C/min. The total program lasted 8 minutes. The calibration curve to measure O_2_ was made using different volumes of air containing different ppm of O_2_ in triplicate. The correlation coefficient of the calibration R^2^ was 0.995.

### Reagents

The Vybrant™ DiD Cell-Labeling Solution (Catalog number: V22887, Invitrogen, Life Technologies), SYTO 9 (Catalog number: S34854, Invitrogen, Life Technologies) and SYTOX Orange (Catalog number: S34861) were received from Thermo Fisher Scientific. Primary stock solutions of Isoniazid (INH), Streptomycin (STR) and Ciprofloxacin (CIP) were made at 40 mg/mL, 100 mg/mL and 30 mg/mL in water respectively. For Rifampicin (RIF) and Metronidazole, 100% dimethyl sulfoxide (DMSO) was used to make 50 mg/mL stock. These were frozen in aliquots at 20°C. At the point of use, the stocks were diluted to the desired concentration with water and then filter sterilized (pore size, 0.2 µm), or in DMSO.

### Cell staining

Bacterial cells are suspended at the density of 1* 10^6^ cells/ mL in their respective growth media. For staining Cg, BHI medium was used, whereas for Bs, Pa, Ec and Sa, LB medium was used. Since, presence of serum could affect staining efficacy as per manufacture’s recommendation, for mycobacterial staining step 7H9 medium containing 0.05% Tween-80, 0.2% glucose and 0.085% NaCl was used. Cells were stained with 5 µM of Vybrant DiD solution for 20 min at 37 °C under shaking condition in dark. An unstained and ethanol treated (vehicle) sample were handled similarly to provide a control. After incubation, cells were washed twice with growth medium to remove remaining dye by spinning them down at 4000 RPM for 5 min. The cells were then resuspended in fresh filtered growth medium. At each time point of flow cytometry, the sample was taken out and stained with 1 µM of nuclear counterstain SYTO 9 and 100 nM of viability counterstain SYTOX Orange for 20 min at room temperature. The sample was analyzed without washing or fixing in flow cytometry. Vybrant DiD, SYTO 9 and SYTOX Orange were detected by filters of 675/25 nm, 533/30 nm and 585/40 nm respectively.

To aid with the gating strategy, the further controls were included. The cells which were either unstained, only SYTO 9 stained cells or dual staining with SYTO 9 and Vybrant DiD enabled the placement of gates, to select the cells from debris. To gate the live cells, controls with heat killed bacteria (80–90 °C for 10 min) that were either unstained, stained with SYTOX Orange or dual stained with SYTOX Orange or Vybrant DiD were used. This enabled the placement of gates, to select the live cells that were Vybrant DiD positive. These controls also helped to verify that there is not spillover of fluorescence from these three stain into their respective detector channels mentioned above.

### Flow cytometry sample preparation, acquisition and analysis

Flow cytometry was performed on a BD Accuri C6 (BD Biosciences, San Jose, CA, US) and data acquisition with BD Accuri C6 software including recording processed sample volume. Manual and extended cleaning cycles were performed at the beginning and end of each flow cytometry session with verification of low event rate in filtered MiliQ water before each run. For reliable cell counts, the events measured by flow cytometer was kept <10^6^ cells/µL of measured sample. This was to ensure that the amount of cells was high enough in comparison to machine noise and low enough to not cause over-saturation of machine. The threshold was set 5000 for forward scatter (FSC) and 500 for the side scatter (SSC). In addition to FSC and SSC, SYTO 9, SYTOX Orange and Vybrant DiD fluorescence intensity was captured using FL1 (533/30 nm), FL2 (585/40 nm) and FL4 (675/25 nm) filters respectively.

Flow cytometry data were analysed using FlowJo vX.0.07r2 software. A primary gate was set based on FSC/SSC properties, following which the SYTO 9 positive (only cells excluding debris) population was gated, within which population with SYTOX Orange (live) was gated. To determine the thresholds to separate live- and dead-stained cells a calibration curve was prepared with mixtures of living and heat-killed cells. The live cells population was then analysed to determine the mean intensity of the Vybrant DiD fluorescence intensity. To calculate the number of generations, based on fluorescence intensity data, the extent of bacterial replication (RF = fold replication) was first determined by the ratio F_0_/F_t_, where F is the mean intensity of Vybrant DiD fluorescence at a specific time. The number of generations was in turn calculated from the formula RF = 2^N^. The number of generations as determined by OD measurements or CFU data was calculated similarly, except that in this case RF = F_t_/F_o_. Generation times are expressed as mean ±SD.

### Time-kill assay

To assess bacterial growth and survival after antibiotic treatment, cultures were diluted to OD = 0.01.The tested concentrations of different antibiotics were then added to the culture and incubated at 37 °C under shaking conditions. At the indicated antibiotic exposure time, the samples were collected and serially diluted (10-fold, 10^0^–10^−6^) and spotted on 7H9-ADC agar plates supplemented with 0.4% activated charcoal. The plates were incubated for 3–4 days at 37 °C to determine surviving CFU counts. For low oxygen culture, the antibiotics were added to culture using injection and treated for 48 hr.

### Antimicrobial treatment and regrowth of antibiotic tolerance cells experiment

After 18 h of exposure to different antibiotics and cells were stained with Vybrant DiD as described above. Cells not exposed to antibiotic were used as untreated control. Subsequently, treated samples and the control were inoculated into fresh 7H9+ADC medium while the negative control was inoculated into PBS containing STR (0.75 µg/mL). All cultures were incubated for 24 h. At different time points, samples was taken out for fluorescence intensity measurement at various time points.

### Macrophage infection experiment

Murine macrophage RAW264.7 cells were cultured in Dulbeco’s Modified Eagle’s Medium (DMEM), supplemented with 10% heat-inactivated fetal bovine serum (FBS) at 37 °C in 5% CO_2_. Cells were passaged every 2–4 days. For infections, cells were seeded at 5 *10^4^ cells per well in 24-well plates. Where required for microscopy, 14 mm glass coverslips were sterilized and added to appropriate wells prior to seeding. When cells were semi-confluent, medium was replaced with fresh DMEM/10% FBS. To prepare mycobacteria for infection, Msm-DsRed was grown till mid-log phase (~0.5 OD) and stained with Vybrant DiD. Bacteria were then washed gently with PBS and suspended in it. The suspension of Vybrant- DiD stained Msm-DsRed were added to macrophages at a MOI of 7: 1, and incubated at 37 °C in 5% CO_2_. After 3 h of uptake period, cells were washed once with PBS before the medium was replaced with DMEM/10% FCS containing 100U penicillin/streptomycin. The cells were then incubated at 37 °C in 5% CO2 for 1 h to kill any non-phagocytosed, extracellular bacteria. Cells were washed three times with PBS before adding fresh DMEM/10% FCS. Time course measurements evaluating fluorescence dilution of Vybrant DiD attributed to bacterial replication were done at 0, 12, 24, 36 and 48 h.

### Fluorescence microscopy

For static imaging of bacteria from in vitro culture medium a drop of sample (∼2 µL) was taken directly from the culture and spotted onto a 1% agarose pad. The samples were allowed to get adsorbed onto the pad. The pad was placed into the ibidi microscope slide and imaged.

For imaging infected macrophages, the coverslips containing them were fixed with 4% formaldehyde overnight at 4 °C, washed with PBS and mounted onto clean microscope slides using ProLong Diamond + DAPI (Invitrogen). Olympus BX63 epifluorescence microscope equipped with Ultrasonic stage and UPLSAPO 100 × 1.4NA oil-immersion objective (Olympus), Lumencore SOLA FISH light source, a Hamamatsu ORCA- Fusion sCMOS camera (6.5 μm pixel size) mounted using U-CMT C-Mount Adapter was used. Samples were imaged using 651–670 nm (for Vybrant DiD), 555–569 nm (for dsRed) and 359–457 nm (for DAPI) filters with exposure time of 350, 350 and 65 ms, respectively. Z-sections were acquired at 300 nm intervals over an optical range of 6.0 µm. For imaging, Vybrant DiD stained different bacteria, exposure time was 10 ms. The cellSens software (Olympus) was used for acquisition.

### Single cell time-lapse microscopy

For time-lapse microscopy, the agarose pads were prepared using low-melting point agarose (1.5% (w/v)) dissolved in 7H9-0.05% Tween-80 media. After agarose was cooled down to around 40–50 °C, 10% of ADC enrichment was added. Gently, 700 µL of agarose was dispensed onto the glass cover slip (18*18 mm) placed in sterile petri dish on the flat surface. The second cover clip was placed on the top to create an agarose sandwich. The petri dish was closed and left for ~30 min to let the agarose solidify. The upper coverslip was then removed gently using a sterile scalpel blade and the required size of agarose pad was cut out. Then, the 2 µL of bacterial sample (~4000 cells/ µL) was pipetted on top of the pad. After allowing cells to get adsorbed for 5–10 min, the agarose pad was flipped onto the glass slide. To image multiple samples at the same time, the glass slide with multiple wells (ibidi) was used and several agarose pads were flipped onto the slide. The lid of the slide was closed and sealed with parafilm to minimize drying of agarose pads. A drop of immersion oil was placed on the 100X objective and the slide was mounted on the microscope stage. The imaging was carried out in time-lapse manner for 120 timeframes each time step of 15 minutes with exposure of 10 ms using a Nikon Ti-eclipse microscope (Nikon, Minato, Tokio, Japan) at 37°C equipped with a TuCamsystem (Andor, Belfast, Northern Ireland) and 2 Andor Zyla 5.5 sCMOS Cameras (Andor) and a SOLA6-LCR-SB light source (Lumencor, Beaverton, OR).

### Image analysis

To determine the effects of hypoxia duration on bacterial resuscitation, the time till first division of single bacteria and colony growth rate were extracted from the single cell time-lapse microscopy data. For determining single colony growth rate, firstly a single image was classified into colony and background pixels using the random-forest classifier present in the Napari-buds plugin [22].The created random forest classification model was saved and applied to all images for batch classification. Next, the function label from scipy.nd image was applied to segment single colony labels per time step. Then, single-colonies were tracked across the time dimension using the btrack library [23]. Segmented images were manually corrected in the Napari viewer. If neighboring colonies merged, these colonies were excluded from the analysis from the point of merger. The increase in area per colony over time was used to determine the growth rate using the first derivative of the Savitsky golay filter from scipy.signal library. In order to determine time till first division, for each cell present at time point zero this event was annotated using the napari point layer. Next, time till first division was extracted and linked to growth rate per colony using the scikit-image processing library python and custom jupyter notebooks [22]. These jupyter notebooks can be found on “https://github.com/SanderSMFISH/Vybrant-DiD-staining-of-mycobacterial-cells-analysis/tree/main/Figure6E_Hypoxia_resurcitation”. To determine bacterial growth and non-growing population size during macrophage infection, intensity of the Vybrant DiD dye was determined within Msm-DsRed positive single bacterial cells. Single cell labels were manually annotated in 3D in the napari viewer. Extraction of fluorescent intensity per cell mask was performed using the scikit-image processing library and custom python notebooks. Cells with an intensity above 4000 were deemed Msm-DsRed and Vybrant DiD positive. These notebooks can be found on “https://github.com/SanderSMFISH/Vybrant-DiD-staining-of-mycobacterial-cells-analysis/tree/main/Figure3_Macrophage_infection”.

## Results

### A fluorescent organic dye for rapid and selective detection of mycobacteria

**Fig 1:**
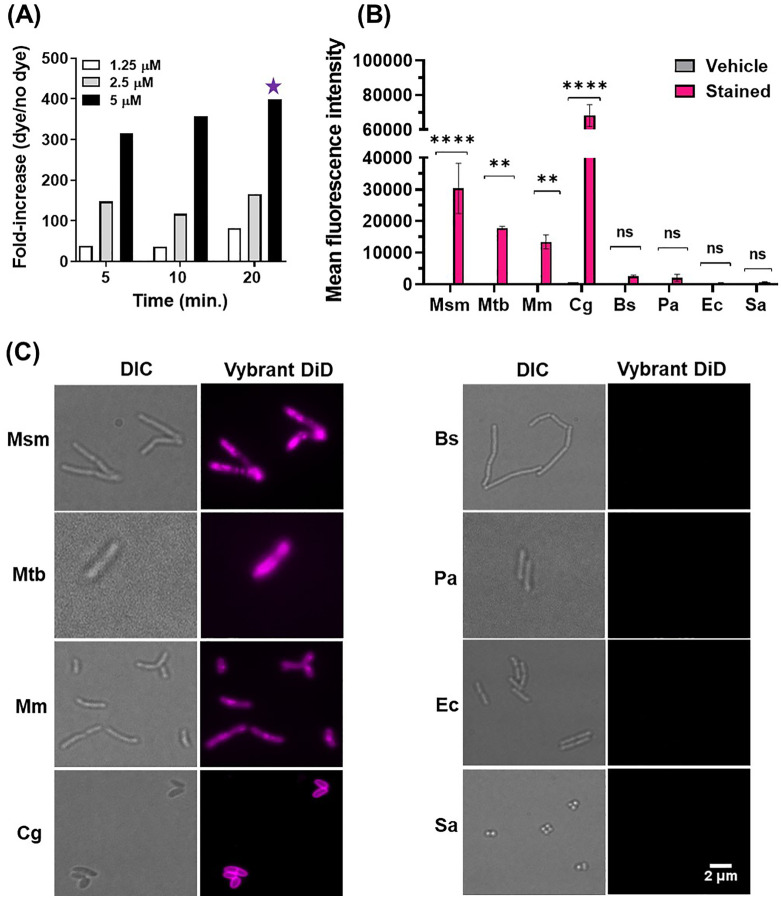
A fluorescent organic dye enables rapid and selective detection of mycobacteria. (A) Flow cytometry analysis of exponentially growing Msm cells (OD_600nm_ ~ 0.4) labeled with various concentrations of dye (1.25, 2.5 and 5 µM) for different duration of time (5, 10 and 20 min). Fluorescence fold-increase (dye/no dye) was calculated by dividing mean fluorescence intensity of stained cells with unlabeled sample. The concentration and incubation time indicated with a star were selected for subsequent experiments. Two biological replicates were performed, one representative replicate is shown. (B) Flow cytometry and **(C)** fluorescence microscopy analysis of live bacterial species *M. smegmatis* (Msm), *M. tuberculosis* (Mtb), *M. marinum* (Mm), *C. glutamicum* (Cg), *B. subtilis* (Bs), *P. aeruginosa* (Pa), *E.coli* (Ec), and *S. aureus* (Sa) labeled with 5 µM Vybrant DiD or vehicle for 20 min. The vehicle (ethanol, dye solvent) sample served as the unlabeled control. 300,000 events in total were analysed in each sample in flow cytometer. Bar graph represents Mean Fluorescence Intensity (MFI) ± Standard Deviation (SD) of at least two independent experiments each with three technical replicates. Data were analysed by two-way ANOVA tests for unequal variances with Sidak’s multiple comparisons test using selected adjusted *p*-values. **p < 0.01, ***p < 0.001, ****p < 0.0001; ns, non-significant. Scale bar, 2 μm.

The hydrophobic cell-envelope is a barrier for labelling mycobacteria with fluorescent dyes [24]. We evaluated two dyes that are currently used for cell staining but to our knowledge have not been extensively used for mycobacteria. Vybrant DiD and CellTracker Deep Red are lipophilic and cell permeable labeling probes respectively (Fig S1A in S1 File), typically used for staining and tracking eukaryotic cells. To explore if these dyes can be used for labeling of live mycobacteria we chose a non-pathogenic and fast-growing member of the *Mycobacterium* genus, *Mycobacterium smegmatis*, which is commonly used as a model bacterium [25]. Whereas *M. smegmatis* stained with CellTracker Deep Red exhibited only a relatively low fluorescence intensity (Fig S1B in S1 File), we observed bright fluorescence of Vybrant DiD labelled cells as assessed by flow cytometry and fluorescence microscopy (Figs 1 and S1B in S1 File). The labeling efficiency of Vybrant DiD was time and concentration dependent, with strong fluorescence increase observed after incubation of 5 min at 2.5 µM Vybrant DiD and a maximum of 400-fold fluorescence increase at 5 µM after 20 minutes incubation when compared to unlabeled cells (Fig 1A).

Based on these results, we continued our studies with Vybrant DiD and extended our tests to pathogenic mycobacterial species. We evaluated labeling of *M. tuberculosis* and *M. marinum* with Vybrant DiD. Indeed, bright labeling was observed for both *M. tuberculosis* and *M. marinum*, which was comparable to *M. smegmatis* (Figs 1B and C). In contrast, other clinically important Gram positive and negative bacteria such *B. subtilis*, *P. aeruginosa*, *E. coli*, and *S. aureus*, exhibited only minimal labeling under similar conditions (Figs 1B and 1C). We hypothesized that the characteristic cell envelope of mycobacteria might be a determinant for efficient labeling (Fig S1C in S1 File). Indeed, *Corynebacterium glutamicum*, a non-mycobacterial member of Actinobacteria phylum with similar cell envelope composition as of mycobacteria, displayed strong labeling by Vybrant DiD dye (Figs 1B and 1C). The variability in fluorescence intensity among Msm, Mma, Mtb, and Cg was determined using raw mean fluorescence intensity (MFI) (Fig 1B). We observed higher mean fluorescence intensity (MFI) for Cg (Fig 1B). In addition, we observed homogenous membrane-associated labelling for Cg, whereas the staining of mycobacterial cells appears more heterogeneous. This variability may reflect differences in envelope architecture or dye distribution within lipid-rich subcellular regions. To correct for fluorescence differences arising from variations in cell size, fluorescence values were normalized to the forward-scatter signal (FSC-A), which is proportional to cell size. After normalization (Fig S1D S1 File), mycobacterial cells displayed the highest fluorescence per optical unit, whereas Cg exhibited among the lowest values, reflecting a lower labeling density per optical size in line with its shorter mycolic acids [26].

These findings indicate that labeling with Vybrant DiD is a rapid, efficient and selective tool for visualizing mycobacteria and other Actinobacteria with both flow cytometry and fluorescence microscopy.

### Quantitative assessment of mycobacterial growth *in vitro*

**Fig 2:**
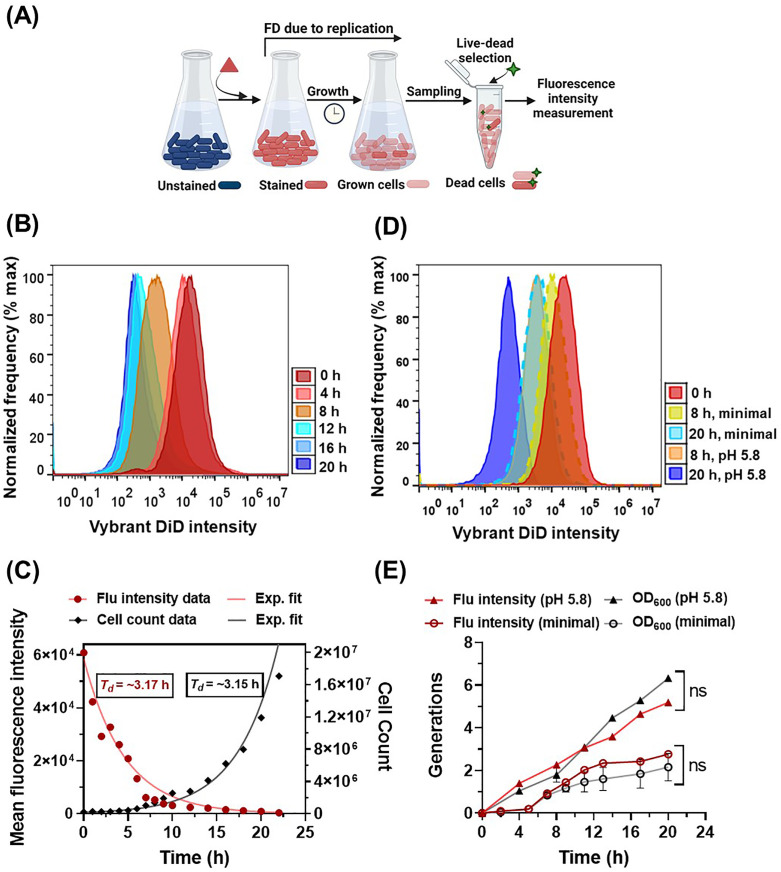
Fluorescence dilution assay using Vybrant DiD to assess mycobacterial replication dynamics under various *in vitro* conditions. (A) Schematic representation of Vybrant DiD based fluorescence dilution (FD) assay to monitor replication dynamics: *M. smegmatis* cells are labeled with Vybrant DiD, gently washed and put into fresh growth media, and as cells grow and replicate the total fluorescence gets diluted with each successive division. The fluorescence intensity of cells is measured using flow cytometry by sampling the cells at different time points. SYTO 9 and SYTOX Orange are used to select live cells by excluding dead cells and media debris. (B) Flow cytometry analysis of Vybrant DiD fluorescence (Flu) intensity at selected time points and **(C)** comparison of generation time calculated from fluorescence intensity (red line) and other conventional methods such as cell count (black line), OD_600nm_ and CFU (table) in 7H9 growth (+ADC, rich) media. The data are representative of three independent experiments. (D) Represents the fluorescence distribution of Vybrant DiD intensity under minimal nutrient (PBS + 0.05% Tween-80) and low pH (5.8 pH) conditions at selected time point and **(E)** compares the bacterial generation numbers calculated from fluorescence intensity (red) and OD_600nm_ (black) measurement under minimal nutrient and low pH conditions. Statistical significance was assessed using a paired two-tailed *t*-test across all corresponding time points (GraphPad Prism 10). 300K events in total were analysed in each sample. The data representative of at least three independent experiments.

Fluorescent probes can be exploited to measure growth of cell populations and identify subpopulations with deviating growth properties [27]. We developed a flow-cytometry based fluorescence-dilution method for quantitative assessment of growth of *M. smegmatis* cells using Vybrant DiD labeling (Fig 2A). The underlying principle is that a labeled mother cell gives rise to two daughter cells after division that each inherit (on average) 50% of the probe of the mother cell. If a population of cells consists of subpopulations with deviating growth rates then this is reflected by the coexistence of cells that differ in their labeling intensity after a given number of generations. Dyes and probes, especially when used in high concentration, are often toxic to live cells, restricting their use to static analysis [28]. Therefore, we first assessed the impact of Vybrant DiD on *M. smegmatis* and found no difference in number of colony forming units (Fig S2A S1 File) and growth (Fig S2B in S1 File) prior and after staining. Vybrant DiD could readily be combined with SYTO 9, a nucleic acid staining dye (Fig S3A S1 File), and SYTOX orange, a dye staining dead or membrane-permeabilized cells (Figs S3B-C in S1 File), facilitating the exclusion of cellular and media debris and membrane-permeabilized/dead cells from the analysis (Fig S3D in S1 File). To ensure that the intensity is predominantly reduced by cell division rather than by diffusive loss of the probe into growth medium, we monitored the fluorescence intensity of Vybrant DiD-labeled *M. smegmatis* cells in a non-replicating, nutrient-limited culture in the presence of a bacteriostatic concentration of streptomycin (STR) for 20 h (Fig S4A in S1 File). Subsequent analysis by flow cytometry showed that the non-replicating streptomycin-treated cells had retained high levels of fluorescence intensity (Fig S4A in S1 File). An initial fluorescence intensity decrease due unspecific dye loss was observed, after which the intensity remained constant (Fig S4B in S1 File). To account for this loss, we fitted the initial fluorescence decay to an exponential decay function and obtained a decay rate constant (k_decay_) describing this passive loss. This constant was then used in all subsequent calculations of growth rates and subpopulation sizes to correct the apparent fluorescence decrease by subtracting k_decay_ from the measured apparent growth rate (Fig S4B in S1 File). These results indicate that the labeling with Vybrant DiD is sufficiently stable to be used as a reporter for mycobacterial cell-growth.

Next, after labeling *M. smegmatis* cells with Vybrant DiD, the dilution of fluorescence in bacteria undergoing active replication in fresh rich growth media was analyzed by flow cytometry at regular time intervals. In parallel, we took samples for OD and Colony forming Unit (CFU) measurements to allow for a comparison of growth rate estimates from these three different methods. The resulting flow cytometry data revealed a decrease in mean fluorescence intensity over time, reflecting fluorescence dilution in the growing cell population (Fig 2B). We calculated the generation time from the mean fluorescence intensities (MFI) over a time course of 22 h (Fig 2C). The generation time obtained from Vybrant DiD fluorescence dilution, ~ 3 h, proved comparable with generation times determined by cell count, OD_600_ measurements and CFU counting (Fig 2C and S2C in S1 File ). Thus, fluorescence-dilution based on Vybrant DiD is a reliable method for the determination of the generation time of a growing population of mycobacterial cells.

Mycobacterial species may encounter diverse fluctuating conditions such as hypoxia, low pH and nutrient limitation inside the host or in the external environment [29]. Such conditions are often applied in *in vitro* experiments to study adaptive responses [30]. We exploited Vybrant DiD to study *M. smegmatis’* growth-response dynamics to low pH and to minimal nutrient condition (Fig 2D). The number of generations calculated for both low pH (division time ~3.3 h; growth rate of 0.30 h^-1^) and minimal nutrient conditions (division time of ~7.24 hours; growth rate of 0.096 h^-1^) correlated well between the fluorescence-dilution based method and OD_600_ measurement for up to approximately six generations (Fig 2E). Control experiments confirmed that the fluorescence intensity of Vybrant DiD *per se* in the absence of bacteria exhibited negligible differences under low pH and minimal nutrient conditions (Fig S4 C). Together, these results demonstrate that fluorescence dilution in combination with Vybrant DiD can be used to study growth dynamics of mycobacteria under various relevant environmental conditions.

### Membrane-labeling method highlights the heterogeneous replication of mycobacteria in a macrophage model

**Fig 3:**
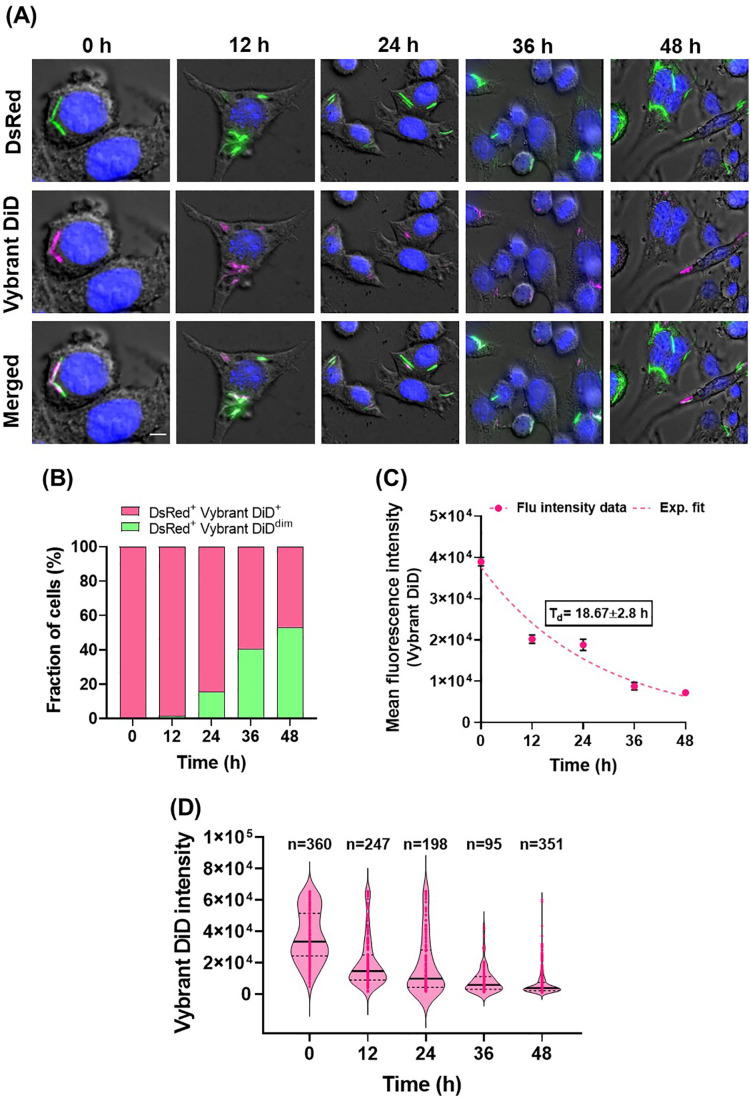
Fluorescence microscopy based visualization and measurement of intracellular mycobacterial replication. (A) Fluorescence microscopy images of RAW264.7 macrophages infected with Vybrant DiD labeled *M. smegmatis* cells to monitor the intracellular replication of bacteria by following the intensity of Vybrant DiD over time. The fluorescence of constitutive expressed DsRed protein is indicative of viable status of cells inside macrophages. The images shows DAPI staining in blue. Scale bar 2 µm. (B) As bacteria replicates inside macrophage, the intensity of Vybrant DiD per bacterial cell decreases. The bar graph representation of fraction of live bacterial cells with high (DsRed^+^ Vybrant DiD^+^, pink bars) and low (DsRed^+^ Vybrant DiD^dim^, green bars) intensity of Vybrant DiD fluorescence over time. (C) Generation time inside macrophages calculated based on the mean fluorescence intensity of Vybrant DiD of live (DsRed^+^) bacterial cells over time, representing the average fluorescence decay kinetics. (D) Represents the Vybrant DiD intensity of individual bacterial cells over time suggestive of heterogeneous nature of replication of mycobacteria inside macrophages. Each dot represents an individual cell. The violin plots show the median (centre line) and interquartile range (dashed lines). Data shown are representative of two independent biological replicates.

Several pathogens, including *M. tuberculosis*, can survive and replicate within human host cells [31,32]. *M. tuberculosis* survival in macrophages has been associated with the recalcitrance to antibiotic treatment and recurrence of infection [33]. Convenient tools that can track the replication and survival of mycobacterial cells in a single macrophage cell could enhance our understanding of host-pathogen interactions and contribute to characterization of novel anti-tuberculosis therapeutics. To assess whether our Vybrant DiD based fluorescence-dilution method allows for the monitoring of mycobacterial replication inside macrophages, we infected mouse RAW 264.7 macrophages with *M. smegmatis* cells labeled using Vybrant DiD. As a marker of viability, *M. smegmatis* cells constitutively expressing DsRed protein [20] were utilized for this experiment (Fig 3A). Analysis by fluorescence microscopy revealed normal morphology of bacteria as well as macrophages, suggesting no toxic effects of Vybrant DiD labeling intracellularly. In addition, the background fluorescence of macrophages remained low during the observation period, suggesting retention of Vybrant DiD within a bacterial population and the absence of any secondary labeling effects of macrophages (Fig 3A). Image analysis demonstrated a gradual decrease in Vybrant DiD fluorescence intensity over time, indicating replication of bacteria inside the macrophages (Fig 3B). The generation time calculated using the mean fluorescence intensity of all Vybrant DiD positive *M. smegmatis* cells within the DsRed-positive (live) population over a period of 48 h was 18.67 ± 2.8 h (Fig 3C). This generation time is similar to values from previously studies using genetically-engineered reporter strains [11] and also indicates minimal replication of intracellular bacteria as compared to *M. smegmatis* grown *in vitro* (Fig 2C).

Based on previous results [4,34], a significant subpopulation of mycobacteria can switch to a non-replicating state in response to stressful conditions, including those associated with the macrophage’s response to infection, making them less sensitive to conventional antibiotics. To address this cellular heterogeneity in mycobacterial replication, we studied the Vybrant DiD intensity profiles of individual bacterial cells inside single macrophages. We found that individual bacteria varied considerably in their fluorescence intensity (Figs 3A and D). Over time, the initial broad distributions of fluorescence intensity of Vybrant DiD became progressively narrower (Figs 3D), reflecting dye dilution through bacterial replication. While most cells showed a gradual decrease in fluorescence, a subset retained high intensity even at later time points, indicating varying replication rates of single bacteria cells (Fig 3D). We conclude that Vybrant DiD based fluorescence dilution method can be exploited for studies of mycobacteria located intracellularly within macrophages, allowing for investigation of mycobacterial replication inside macrophages.

### Quantification of slow-/non-growing subpopulations

Isogenic bacteria often display cell-to-cell heterogeneity of phenotypic properties. Subpopulations of slow-/non-growing cells can coexist with fast-growing cells within the same population [8,35]. In an exponentially growing culture, slow-/non-growing bacteria typically are only a minority subpopulation [6,35–37]. However, due to their slow metabolism and different phenotype, slow-/non-growing subpopulations may have a higher chance for survival in the presence of stresses that impact growing cells, including nutrient limitation, low oxygen tension or antimicrobial treatments [4,8,38]. Bacteria surviving these stressful conditions may subsequently reestablish (disease-causing) populations of coexisting slow-/non-growing and fast-growing cells [3]. Therefore, counting (quantification) of slow-/non-growing bacteria is important for optimization of antibacterial therapies.

**Fig 4:**
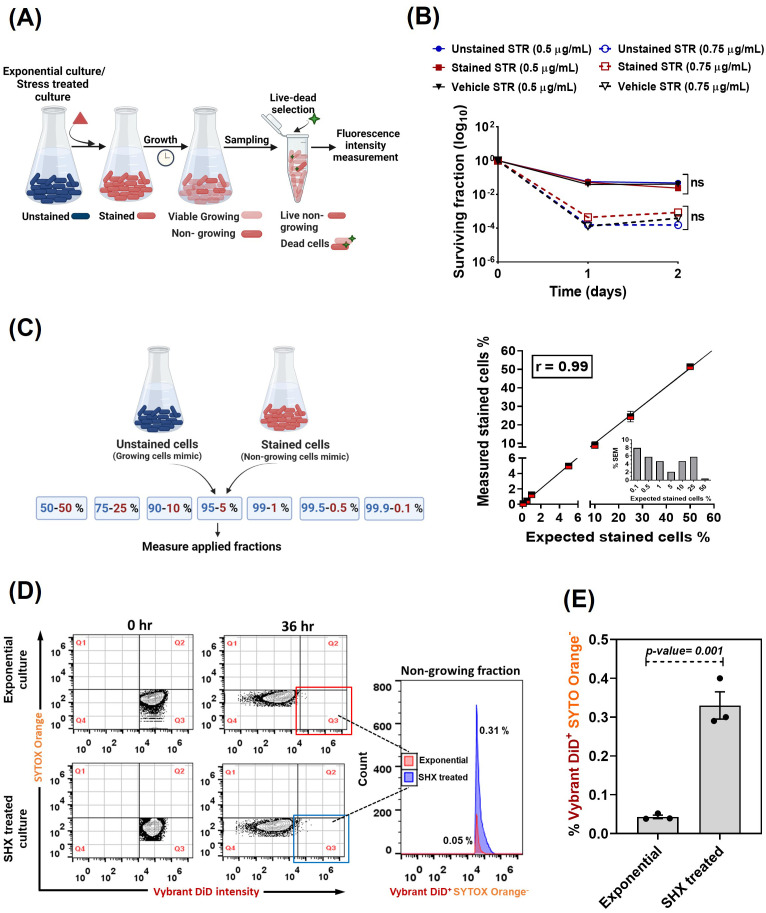
Quantification of slow-/non- growing subpopulation of cells. (A) Using Vybrant DiD-based mycobacterial labeling, slow-/non-growing fraction can be estimated for exponentially growing or stress treated culture: cells are stained with Vybrant DiD, the fluorescence intensity of growing cells dilutes due to division, whereas the slow-/non-growing cells will retain high fluorescence compared to growing cells. This difference in intensity of cells is used to estimate the different fractions using flow cytometry. (B) Biphasic kill curve of exponentially growing *M. smegmatis* cells labeled with Vybrant DiD to evaluate the impact of labeling on the *M. smegmatis* fraction surviving drug treatment.. Cells are exposed to 0.5 and 0.75 ug/mL of Streptomycin (STR). The vehicle sample contains solvent (ethanol) used to dissolve the dye. The unstained sample underwent the washing procedure during staining protocol. (C) Mimic experiment to determine the limit of detection to estimate non-dividing fraction. The unstained cells and Vybrant DiD stained *M. smegmatis* cells mimicking growing and non-growing cells respectively, were mixed in different ratios and the applied fractions were quantified using flow cytometry. The correlation plot at the right represents mean number of cells ± SEM of fractions of at least three independent experiments. Pearson correlation coefficient (r) between expected and measured fractions was 0.99. Inset in plot shows the % Standard error of Mean for each expected fractions. (D) Dot plots and histogram depicting the percentage of live non-growing fraction (Vybrant DiD^+^ SYTOX Orange^-^) in exponentially growing and Serine Hydroxamate (SHX) treated *M. smegmatis* culture. Two independent experiments were done, the data shown are representative of one representative experiment. (E) Bar graph representing the mean ± SEM of percentages of non-growing fraction of three independent experiments each containing at least three technical replicates. The statistical significance is calculated using unpaired two-tailed t test. 3M events in total were analysed in each sample.

We exploited Vybrant DiD based labeling to quantify the slow-/non-growing fraction of *M. smegmatis* by focusing on intact bacteria that retain the dye in a growing population of cells (Fig 4A). We estimated the fraction of slow-/non-growing intact cells in exponentially growing (unstressed) and serine hydroxamate (SHX) stress treated *M. smegmatis* cultures (Fig 4A). SHX is an analogue of L-serine that has mainly been studied in *E. coli*. SHX elicits the stringent response to amino-acid starvation by competitively inhibiting the seryl-tRNA charging enzyme, leading to enhancement of the slow-/non-growing subpopulation [39–41]. We first confirmed that staining or staining procedure *per se* had no significant effect on the fraction of *M. smegmatis* surviving drug treatment, as shown by kill curve analysis of Vybrant DiD-stained and unstained cells using streptomycin as model treatment (Fig 4B). Next, we tested the accuracy of the method by determining the minimum size of a subpopulation of non-growing cells that we could reliably distinguish in a population of cells of which the majority is growing. We mixed unstained and stained cells, representing growing and non-growing populations respectively, in different percentages followed by flow cytometry analysis (Fig 4C). Based on this analysis, the assay demonstrates high precision, reliably detecting non-growing subpopulations as small as 0.1% with less than 10% measurement error (7.97%SEM), and the strong correlation observed (R = 0.99) suggests that even smaller fractions can be detected with comparable accuracy (Fig 4C). Next, we quantified the non-growing fraction in an exponentially growing culture of *M. smegmatis* using stained cells. Initially, all cells showed maximal Vybrant DiD intensity as represented in Quadrant 3 (Q3) (Fig 4D). For growing cells the fluorescence intensity diluted (Q4), while the non-growing fraction retained high fluorescence (Q3, 36 h, Fig 4D). We estimated the percentage non-growing fraction in an exponential culture as 0.05 ± 0.005 (10.49% SEM) (Fig 4D-E). As expected, the percentage non-growing fraction significantly increased in SHX-treated culture to 0.33 ± 0.035 (10.64% SEM) (Fig 4D–E). Together, these results show that Vybrant DiD can be exploited to quantify growth-rate varying subpopulations, associated with persister formation.

### Quantifying regrowth of antibiotic tolerant mycobacterial cells

**Fig 5:**
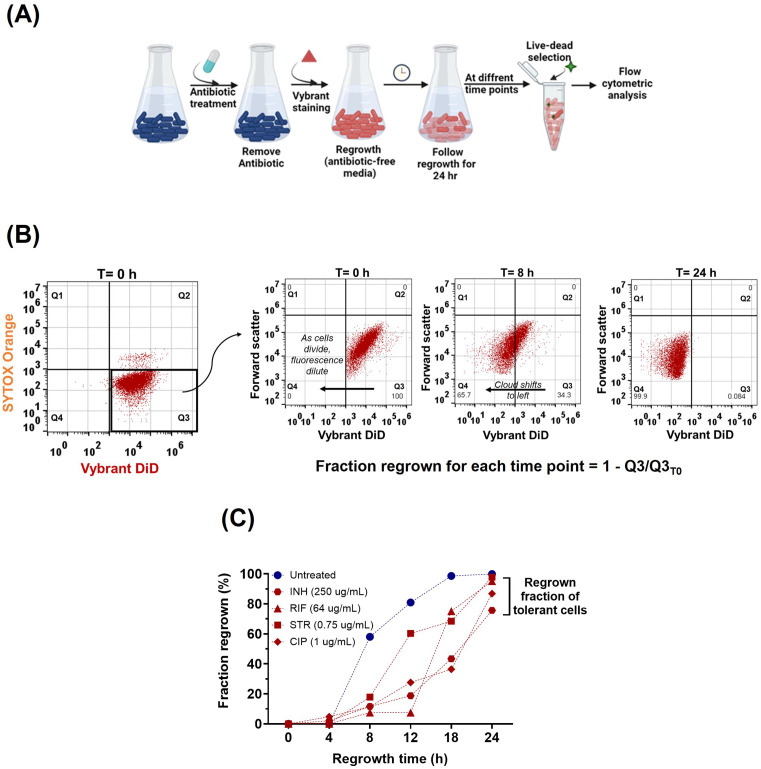
Assessment of regrowth and quantification of the fraction of antibiotic tolerant cells of mycobacteria. (A) Schematic overview of the procedure that involves the treatment of exponentially growing *M. smegmatis* cells with different antibiotics for 16 hours, followed by a gentle wash to remove the antibiotic. The antibiotic-treated cells were stained with Vybrant DiD and regrown in fresh growth media for 24 hr. At different time interval the sample is taken out for flow cytometry analysis (B). Dot plot representation of flow cytometry analysis over time to calculate the fraction of tolerant *M. smegmatis* cells that survived antibiotic treatment. After double staining of INH (250 µg/mL) treated cells, at T = 0 h cells were analysed for Vybrant DiD and SYTOX orange intensity. The live, membrane- stained cells for each selected time point (Q3, bold black box), is further plotted on Vybrant DiD intensity v/s forward scatter area graph to monitor the change in Vybrant intensity of the single cells over time. The Vybrant DiD intensity gradually diluted as the tolerant cells grew and divide. Similar analysis flow was used for other antibiotics. (C) The fraction of regrowing cells for each selected time points of Isoniazid (INH), Rifampicin (RIF), Streptomycin (STR) and Ciprofloxacin (CIP) is calculated for by 1 minus the number of cells in Q3 (of Vybrant DiD intensity v/s forward scatter) at particular time point divided by the number of cells in Q3 at T0. The calculated tolerant fraction is then plotted as percentages. Cells not treated with antibiotic were used as untreated sample. 500K events in total were analysed in each sample.

Next, we utilized the Vybrant DiD dye to quantify the regrowth of bacteria that survived antibiotic treatment, since the ability to resume growth after exposure is a hallmark of cells that were in a tolerant or persister state during treatment (Fig 5A). After treating exponentially growing *M. smegmatis* with standard-of-care tuberculosis antibiotics, the cells were stained with Vybrant DiD, inoculated into fresh growth medium, and subjected to flow cytometry analysis at various time points (Fig 5A). The fraction of regrowing cells (i.e., cells that had been tolerant during antibiotic exposure and subsequently resumed division), for the different antibiotics was calculated by subtracting the number of non-dividing cells (Q3) observed at each time point from the initial number of non-dividing cells (Q3, T = 0h) and then normalizing this difference to the initial non-dividing population. This provides the relative proportion of cells that resumed growth over time (Fig 5B, shows exemplary analysis for isoniazid). For all tested antibiotics, i.e., isoniazid, rifampicin, streptomycin and ciprofloxacin, which target cell wall biosynthesis, transcription, translation and replication respectively, both the fluorescence intensity per cell and number of Vybrant DiD positive cells decreased with culturing time after antibiotic treatment, indicating bacterial division after regrowth of surviving cells (Fig 5B-C and S5 Fig in S1 File). No regrowth was observed in the control sample, where labeled *M. smegmatis* cells were kept in non-replicating, nutrient-limited culture in presence of STR (Fig S5 in S1 File), indicating that the Vybrant DiD fluorescence intensity did not decrease due to any diffusive loss during the experiment. The regrowth of antibiotic treated samples was considerably slower as compared to an untreated sample (Fig 5C and S5 Fig in S1 File), which is due exhibition of lag phase (~8 hours) by the surviving bacteria before resuscitation. The results show that the Vybrant DiD method can be used to quantify bacterial regrowth after drug treatment.

### Quantification of resuscitation of non-replicating persistent mycobacterial cells

**Fig 6:**
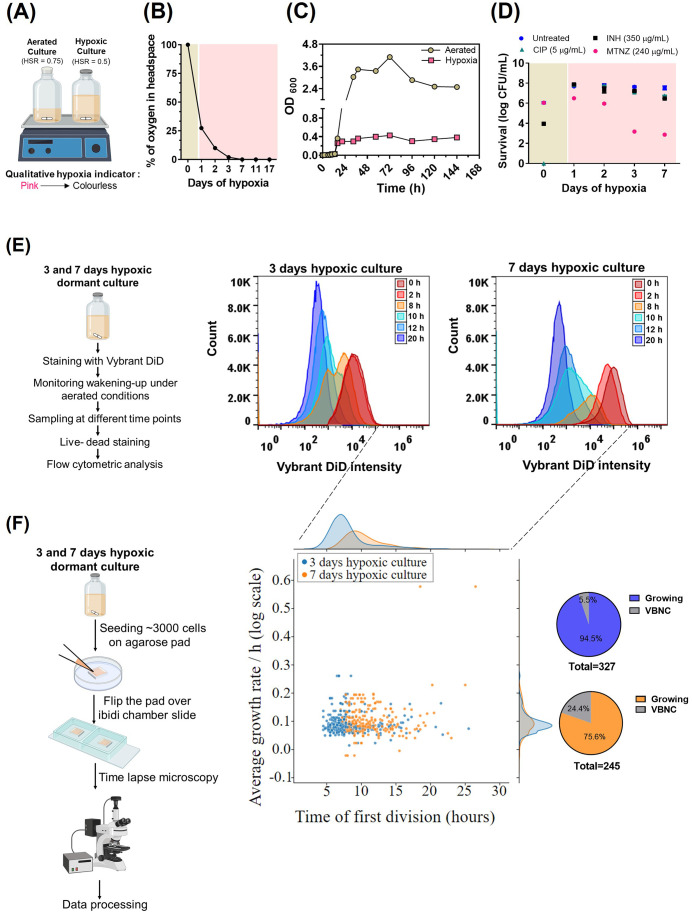
Investigation of resuscitation of non-replicating persistent mycobacterial cells. (A) Schematic of low-oxygen model utilized to generate dormant cells of M. smegmatis. Whereas aerated culture was grown in unsealed glass bottle with 0.75 head space ratio (HSR), low-oxygen culture was grown in sealed glass bottle with 0.5 HSR. The cultures were gently stirred using magnetic stirring bars. Depletion of oxygen was monitored by using a qualitative resazurin anaerobic indicator strip which turns from pink to colourless in low oxygen conditions and (B) Gas chromatography. The brown and pink shading indicates the periods of aerobic and low-oxygen respectively. Mean ± SD of three biological replicates are plotted. (C) Growth of *M. smegmatis* was monitored in aerated and low-oxygen condition by OD_600nm_ at various time points. (D) Antibiotic susceptibility of low-oxygen culture to INH (350 µg/mL), CIP (5 µg/mL) and Metronidazole (MTNZ, 240 µg/mL) after 48 h exposure at indicated days of low-oxygen. The surviving CFU/mL at 0 h (brown shading) indicates the fully aerated condition. The result is representative of three biological replicates. (E) Vybrant DiD-based labeling approach to monitor the resuscitation of hypoxic dormant *M. smegmatis* cells. After 3 and 7 days of hypoxia, the cells were stained with Vybrant DiD and their awakening was followed over time using flow cytometry. 300K events in total were analysed in each sample. The data is representative of two biological experiments. (F) Single-cell time-lapse microscopy to confirm the heterogeneity in resuscitation of hypoxic dormant *M. smegmatis* cells. Distribution and scatterplot of single-cell resuscitation times and growth rate. For 3 and 7 days hypoxic cells n = 327 and n = 245, respectively. The pie charts to the right represent the likely Viable but Non-Culturable cells (VBNC) that did not grow till the last time point of the experiment.

Next, we examined a key factor in mycobacterial physiology and pathogenesis, the resuscitation from a non-replicative state that is associated with recalcitrance to antibiotic treatment. To induce this non-replicative state, we utilized the *in vitro* gradual oxygen-depletion model developed by Wayne [21,42] (Fig 6A). In this model, decline in oxygen over time resulted in cessation of mycobacterial growth at a clearly lower cell density as compared to an aerated culture, attributed to a physiological state referred to as “non-replicating persistence” (Figs 6 B and C, [21]). Consistent with previous data [43], the low-oxygen culture exhibited resistance to both isoniazid and ciprofloxacin, which kill actively replicating bacteria, but were sensitive to metronidazole, which is an anaerobic bactericide (Fig 6D).

To assess the resuscitation of the mycobacteria from the hypoxic culture, we stained *M. smegmatis* cells, which were exposed to oxygen depletion for 3 and 7 days, with Vybrant DiD followed by growth in aerated fresh media. The fluorescence intensity distributions revealed that the apparent homogenous bacterial population diverged into distinct subpopulations of cells after 8 hours of regrowth for both 3 and 7 days of hypoxic culture (Fig 6E). This heterogeneous fluorescence intensity of cells after regrowth is indicative of a varying resuscitation time of cells from their non replicating state. To further zoom-in on these results, we utilized time-lapse microscopy to examine individual non replicating *M. smegmatis* cells and their resuscitation on agarose pads (Fig 6F, S1 Movie). The vast majority of cells (94.5% for 3 days hypoxia and 75.6% for 7 days hypoxia) started dividing within the 30 hours duration of the experiment (Fig 6F). Since we did not observe significant decline in CFU count until 7 days of hypoxia (Fig 6D), the cells that did not start dividing within 30 hours are most likely not dead but could be presumably classified as Viable but Non-Culturable cells (VBNC) (Fig 6F). The distribution of single-cell resuscitation times showed high cell-to-cell variability, ranging from ~5 h to ~25 h for cells from 3-day hypoxic culture and ~10 h to up to ~25 h for cells from 7 day hypoxic culture (Fig 6F, S1 Movie). This finding suggests that with longer exposure to low-oxygen environments, the resuscitation time increases as well. In contrast, the growth rate of individual cells did not correlate to their resuscitation time (Fig 6F), resuscitated cells divided at a rate that did not differ from the growth rate of an aerated culture (~ 0.2 ± 0.0095, [44]). These results show the suitability of this method to study the resuscitation of mycobacteria and its related heterogeneity and its potential to unravel the underlying factors.

## Discussion

Since growth and division are central aspects of bacterial physiology and pathogenesis, accurate and quantitative determination of these parameters is of vital importance in both fundamental microbiology as well as for applications in biomedicine. Monitoring mycobacterial colonies on agar plates or assessing the turbidity of the liquid culture by optical density measurement have been the most commonly utilized methods for assessing mycobacterial growth and replication both *in vitro* or *in vivo* [45]. However, despite being the methodological foundation in mycobacterial research, these techniques are limited by several technical and practical challenges such as time of experimentation, less accuracy and precision, intra- and inter- lab variability, inhomogeneity due to clumping, and uneven spreading leading to neighbor effect [46,47]. In addition, these standard techniques do not provide information on possible phenotypic heterogeneity within the studied bacterial population, e.g., the presence of subpopulations with deviating growth characteristics. Genetically engineered mycobacterial strains, equipped with fluorescent reporters, in combination with flow cytometry and time-lapse microscopy, have facilitated accurate monitoring of mycobacterial growth beyond one generation and have been incorporated in high-throughput screening efforts. However, their use is often limited by the challenges associated with applications in genetically complex and intractable mycobacteria, e.g., in clinical and environmental isolates. Small organic fluorophores, which can be used independent of genetic modifications, have been applied for live/dead staining [48], assessment of antibiotic efficacy [49,50], investigation of cell wall biosynthesis [43,51–53], staining lipid bodies [54], measuring membrane potential [55], metabolic activity [56], DNA content and efflux pump activity [57]. In this study, we report on development of a fluorescent organic lipophilic probe as a tool for assessing mycobacterial growth and division. Our probe is sufficiently bright and stable during the course of an experiment and does not influence physiological properties of bacteria. The growth parameters calculated by our approach, both *in vitro* (under various physiological relevant conditions) and intracellularly in macrophage, closely match those determined by standard methods and corroborate previous studies that used genetically engineered reporters. Due to this observed reliability and accuracy, combined with convenience and utility, we expect broad applications for our approach in replication rate measurements.

We report usage of a small organic fluorophore for rapid, convenient, and selective labeling and detection of mycobacteria. Detection and diagnostics of mycobacteria currently is performed by either culture-based or PCR-based diagnostic testing or by microscopy-based sputum smear test. Culture-based diagnostics is labor-intensive, requiring incubation in specialized facilities for several days to weeks, whereas the PCR-based approach is costly and demands trained expertise. For microscopy-based tests, several fluorophores have been described [58–61], but the most widely used technique remains the acid-fast staining, which involves staining of mycobacteria with either with fluorescent Auramine O dye or Ziehl-Neelsen stain. While it can effectively identify mycobacteria and be applied in low-resource settings, the staining process is harsh. It requires multiple washing steps to remove nonspecific background fluorescence and a rigorous counterstaining procedure to visualize stained bacteria. In this context, our Vybrant DiD based labeling approach has many favorable properties for point-of-care deployment: (i) simple staining procedure, (ii) rapid and selective labeling of live or fixed mycobacteria, (iii) no requirement of uptake or metabolizing of probe by cells, which is affected by cell’s permeability and energy status, (iv) good signal-background ratio, (v) stability at room temperature, (vi) accessible and affordable, and (vii) suitable for detection by flow cytometry and microscopy. We foresee that Vybrant DiD method can be incorporated in high-throughput analysis methods such as high content microscopy and microplate-based flow cytometry. These properties may make this method suitable for diagnostic and characterization of multiple mycobacterial samples, for instances of patient-derived clinical strains.

In the field of mycobacteriology, to quantity and study slow-/non-growing bacteria or bacteria tolerant to antibiotic treatment, typically stress conditions are applied, including hypoxia, low pH, nutrient limitation, treatment with antibacterials or combinations of these factors, so that essentially the whole population consists of slow-/non-growing bacteria [42,62–65]. For growth under standard laboratory conditions the slow-/non- growing subpopulation typically is not quantified. In addition, the few quantitative studies of these phenotypes used genetically modified strains [12,13,66–68]. In our study, we quantified the slow-/non-growing mycobacterial subpopulation during exponential growth of genetically unmodified bacteria. In combination with FACS, our method may also allow for isolation of slow-/non-growing bacterial cells from the population for further downstream analysis. Quantification of such slow-/non-growing subpopulations has been reported earlier for *E. coli* [35], where the fraction persisting during antibiotic treatment under exponentially growing condition accounted for <0.01% of the bacterial population. In our study we found a similar small number of slow-/non-growing cells, 0.05%, for exponentially growing *M. smegmatis*. This small fraction of slow-/non-growing bacteria, in combination with their transient nature, may explain why these persister phenotypes are not accounted for in most experimental settings. We also demonstrated that the slow-/non-growing subpopulation considerably increased upon treatment with SHX. SHX has been extensively used to characterize the stringent response in *E. coli* [39] and has been applied to study cell envelope changes during the stringent response in *Mycobacterium marinum* [69]. However, to our knowledge, no previous reports are available on the impact of SHX on slow-/non-growing subpopulations in mycobacteria. The relation between SHX, the stringent response, slow-/non-growing bacteria and antibiotic tolerance in mycobacteria needs to be further investigated.

Exiting the slow-/non-growing state and reestablishing a growing population upon stress removal is a hallmark of bacterial cells surviving stress and/or antibiotic treatment. Using our exploratory methodology, we monitored and quantified the regrowth of surviving cells after antibiotic treatment and after hypoxia. Usually, a classical plating assay, which determines the CFU, is performed to estimate the number of surviving tolerant cells after cessation of stress. However, as stated above, this technique is laborious, less precise, and shows high experimental variability. Exemplifying these technical challenges, it has been shown that the number of *E. coli* persisters resuscitating on agar plates after ampicillin treatment was found to be significantly lower than that in liquid cultures [70], indicating more precision of flow-cytometry based tools over the standard plating technique. Hence, rapid methods suitable for high-throughput applications, as described previously for assessing tolerance against antibiotics in *E. coli* [70,71], and applied in our study for mycobacteria can be an alternative to assess stress-tolerant fractions, especially in screening drug libraries. We demonstrated that mycobacteria surviving antibiotic treatment exhibit a lag phase before resuscitation in fresh 7H9 media after the removal of the antibiotics. Previously, using mCherry as fluorescent genetic marker, it has been shown that *E. coli* cells surviving ampicillin treatment were able to resuscitate within 1 hour after removal of antibiotic stress, and the doubling time did not differ from untreated cells [70]. The longer resuscitation time determined here for *M. smegmatis* (3–4 hours) may be reflective of the longer generation time of mycobacteria as compared to *E. coli*. Arguably, the regrowth of cells within the short experimental period indicates that resistance mutations are unlikely to account for the observed phenotype. Such mutations are rare (e.g., 1 in 10^8–10^9 cells) and do not confer an immediate growth-rate advantage under these conditions. Therefore, it is most likely that the observed regrowth is due to a transient state of insusceptibility to the antibiotic associated with slow growth rather than a heritable resistance mutation. However, we did not sequence these strains.

We have also shown that Vybrant DiD could be utilized to study the dynamics of mycobacterial resuscitation from hypoxia, arguably the most widely applied *in vitro* model for non-replicating persistence. While a wealth of studies is available on induction of a slow-/non-growing state, reports examining resuscitation from this state are scarce. Our results show that after a period of dormancy, resuscitation of cells is heterogeneous and stochastic. In addition, the length of hypoxic stress also impacts the duration of resuscitation. Similarly, resuscitation of *E. coli* was distributed over time after antibiotic treatment [72], and longer stationary phase delayed the growth resumption [73]. Apparently, irrespective of the type of stress employed, the resuscitation characteristics are similar between two organisms.

The observed heterogeneity in the timing of growth resumption has been attributed to a bet-hedging strategy where bacteria with short lag phase can make the most of the new resources available, whereas the cells that remain in a slow-/non-growing state for longer insure against possible hostile conditions [35]. Another model that has been suggested is the scout model [74], which hypothesizes that independent of environmental cues, time to time, a cell, called scout, stochastically recovers from dormancy and scans its surroundings for the opportunity to grow, while the rest of a population remains slow-/non-growing. This random, low-frequency exit from the slow-/non-growing state has been shown for environmental isolates, *E. coli* and *M. smegmatis* suggesting that this may be a general microbial survival strategy [75]. Convenient and reliable probes, as developed and described in our report, may be instrumental for testing the above hypotheses and for the investigation of cellular and molecular factors underlying formation of bacterial cells that can persist under adverse conditions as well as their resuscitation.

In summary, our data validate the Vybrant DiD based labeling as a rapid method for *in vitro* and intracellular labeling of mycobacteria, which may be applied in basic research, drug discovery or medical diagnostics. Labeling with Vybrant DiD may be extended to pathogenic mycobacterial species, or mycobacteria obtained from environmental isolates, which are difficult to genetically modify due to their less known biology. In addition, Vybrant DiD may also be utilized for efficient labeling, detection or characterization of *Corynebacterium*, a bacterium widely used in biotechnology applications, potentially in industrial settings.

### Limitations of the study

In this study, the methodology developed offers a simple yet highly quantitative approach to estimate slow/non-growing mycobacteria *in vitro* and intracellular, it also has limitations. Although, Vybrant DiD is a lipophilic dye expected to intercalate into lipid membranes, we have not yet determined which cellular components are stained by Vybrant DiD and whether the dye stains the same components across different bacteria species. Since this approach is based on dilution of dye, we can only study slow-/non-growing bacterial cells formed initially, not those formed during the cell divisions throughout the experiment, as their fluorescence would be considerably low. Additionally, to further advance this method for point- of-care detection, testing it for clinical strains of mycobacteria would be interesting for future research. For using this dye on patient-derived clinical strains or sputum samples, the lipophilic nature of the dye and the heterogeneous nature of such samples (often enriched in dead tissue and dead cell aggregates), may need more standardization of staining method and optimization of the dye. Moreover, combining this method with time-lapse microscopy and advanced data analysis tools, this approach can also help in a lineage study of slow-/non-growing subpopulations, aiding in understanding when and how individual cells enter and exit a slow-/non-growing state.

## Supporting information

S1 File Supplementary Figures. (PPTX)

S2 File Minimal data set. (PDF)

S1 Movie Time lapse movie of *M. smegmatis* cells showing resuscitation after hypoxia stress. Images were taken at 15 minute interval for 30 hours.
(AVI)
