# Supplementary material for: Rapid and efficient labeling by a selective organic fluorophore probe highlights heterogeneity of mycobacterial populations and persister resuscitation
Source: PLoS One. 2025 Dec 18;20(12):e0338563. doi: 10.1371/journal.pone.0338563 (PMC12714215; doi:10.1371/journal.pone.0338563)
Supplement: S1 File — (PPTX) [file pone.0338563.s001.pptx]

## Slide 1
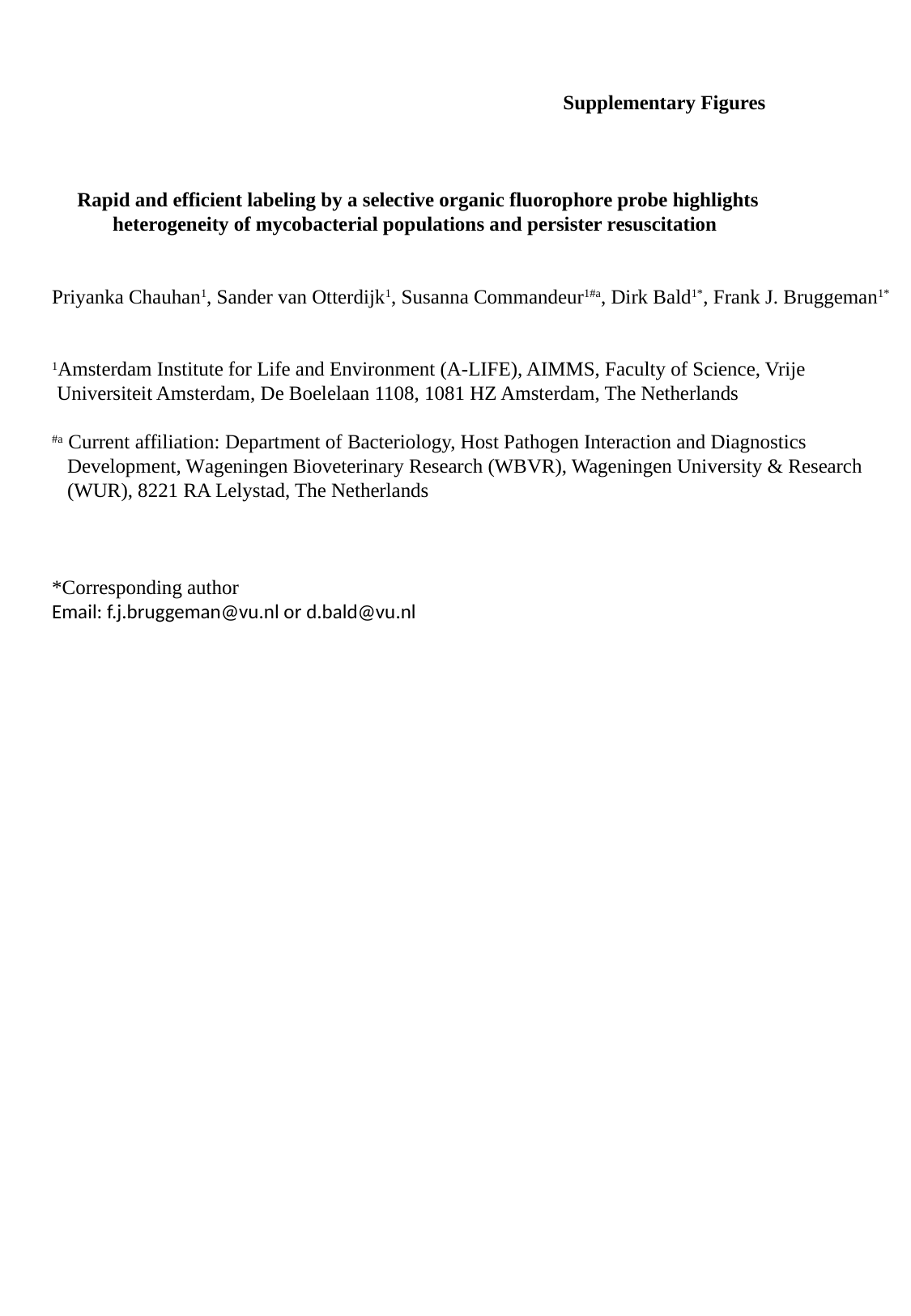

Supplementary Figures
 Rapid and efficient labeling by a selective organic fluorophore probe highlights
 heterogeneity of mycobacterial populations and persister resuscitation
Priyanka Chauhan1, Sander van Otterdijk1, Susanna Commandeur1#a, Dirk Bald1*, Frank J. Bruggeman1*
1Amsterdam Institute for Life and Environment (A-LIFE), AIMMS, Faculty of Science, Vrije
 Universiteit Amsterdam, De Boelelaan 1108, 1081 HZ Amsterdam, The Netherlands
#a Current affiliation: Department of Bacteriology, Host Pathogen Interaction and Diagnostics
 Development, Wageningen Bioveterinary Research (WBVR), Wageningen University & Research
 (WUR), 8221 RA Lelystad, The Netherlands
*Corresponding author
Email: f.j.bruggeman@vu.nl or d.bald@vu.nl

## Slide 2
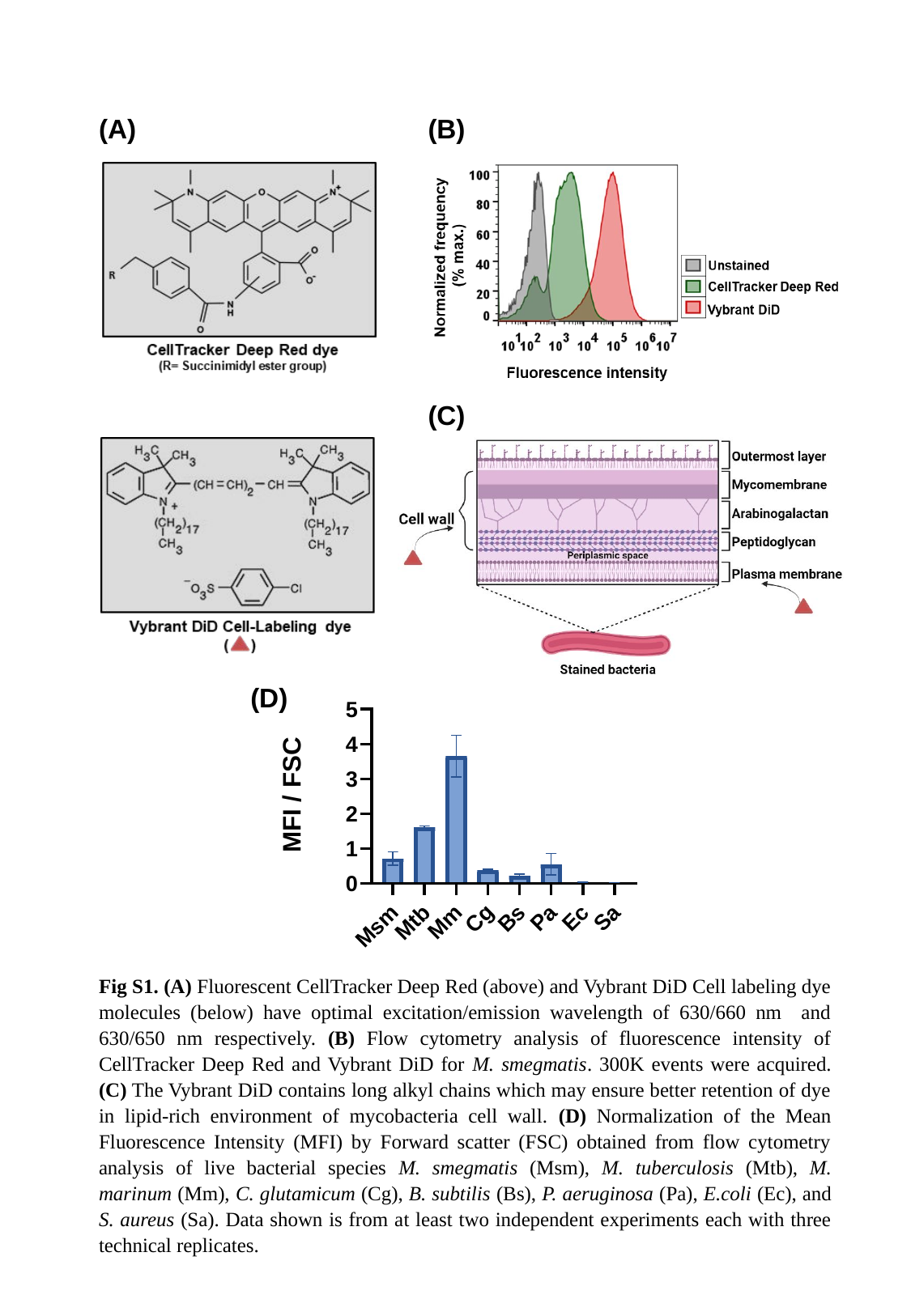

(A)
(B)
(C)
(D)
Fig S1. (A) Fluorescent CellTracker Deep Red (above) and Vybrant DiD Cell labeling dye molecules (below) have optimal excitation/emission wavelength of 630/660 nm and 630/650 nm respectively. (B) Flow cytometry analysis of fluorescence intensity of CellTracker Deep Red and Vybrant DiD for M. smegmatis. 300K events were acquired. (C) The Vybrant DiD contains long alkyl chains which may ensure better retention of dye in lipid-rich environment of mycobacteria cell wall. (D) Normalization of the Mean Fluorescence Intensity (MFI) by Forward scatter (FSC) obtained from flow cytometry analysis of live bacterial species M. smegmatis (Msm), M. tuberculosis (Mtb), M. marinum (Mm), C. glutamicum (Cg), B. subtilis (Bs), P. aeruginosa (Pa), E.coli (Ec), and S. aureus (Sa). Data shown is from at least two independent experiments each with three technical replicates.

## Slide 3
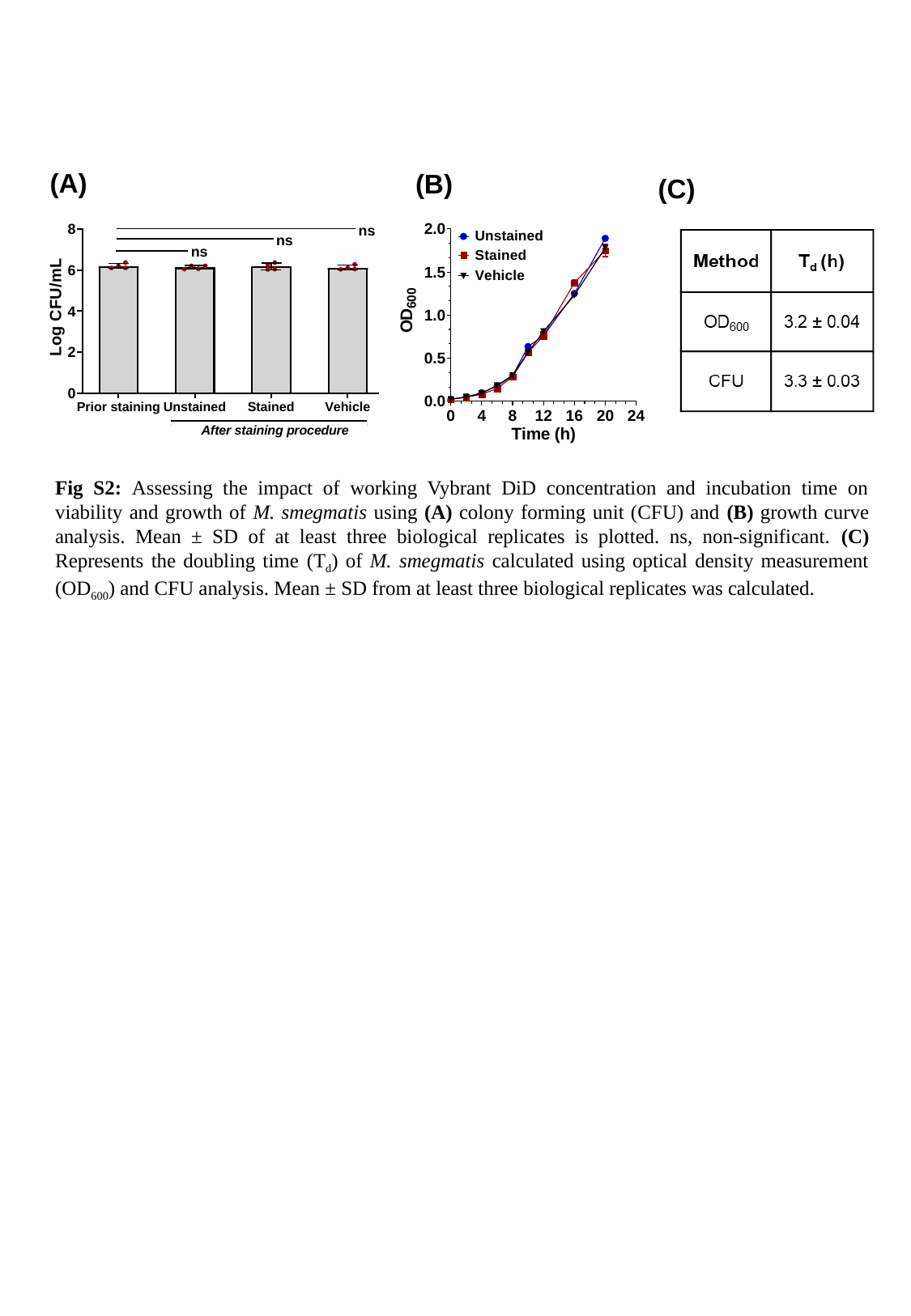

(A)
(B)
(C)
Fig S2: Assessing the impact of working Vybrant DiD concentration and incubation time on viability and growth of M. smegmatis using (A) colony forming unit (CFU) and (B) growth curve analysis. Mean ± SD of at least three biological replicates is plotted. ns, non-significant. (C) Represents the doubling time (Td) of M. smegmatis calculated using optical density measurement (OD600) and CFU analysis. Mean ± SD from at least three biological replicates was calculated.

## Slide 4
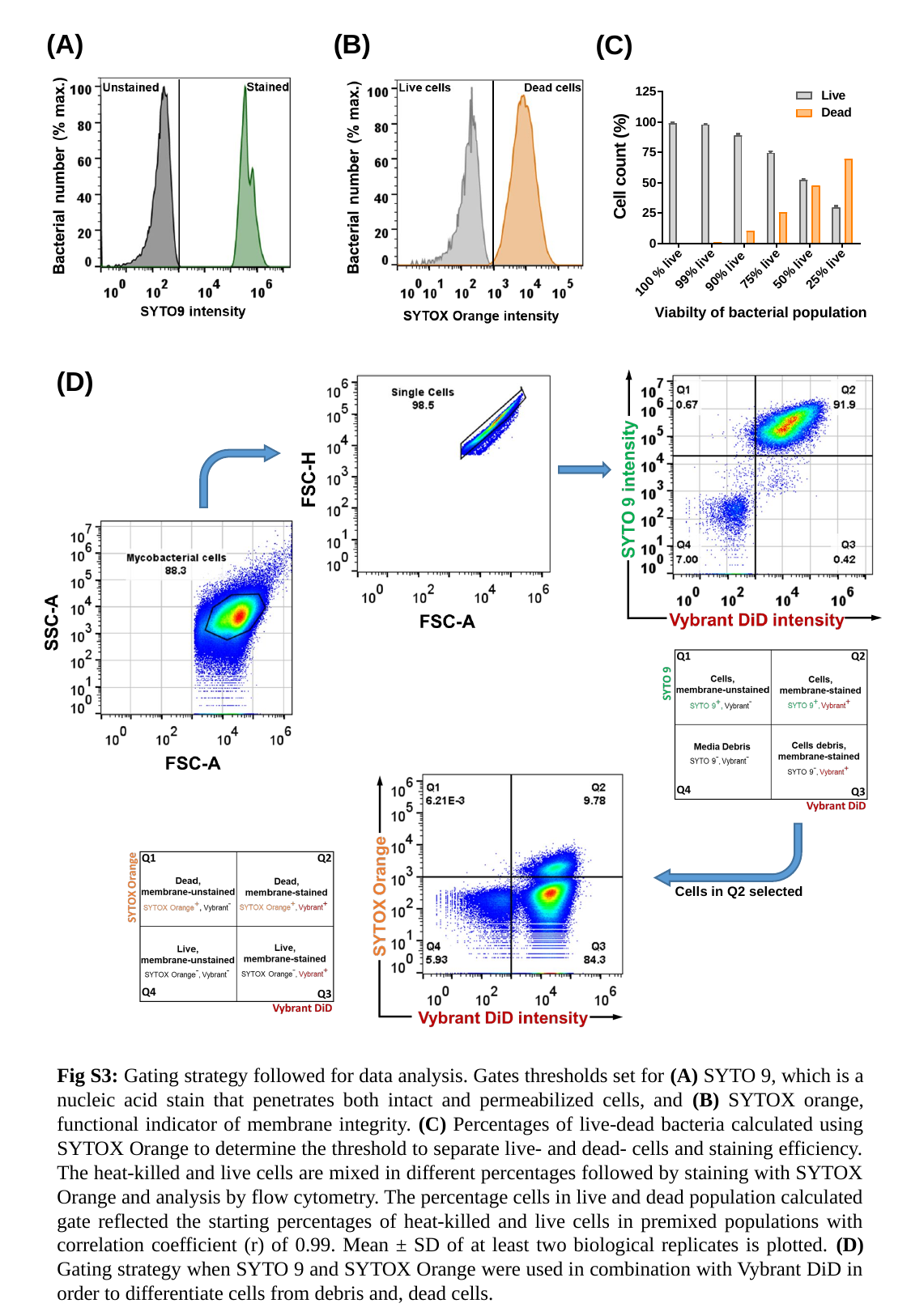

(B)
(A)
(C)
(D)
Cells in Q2 selected
Fig S3: Gating strategy followed for data analysis. Gates thresholds set for (A) SYTO 9, which is a nucleic acid stain that penetrates both intact and permeabilized cells, and (B) SYTOX orange, functional indicator of membrane integrity. (C) Percentages of live-dead bacteria calculated using SYTOX Orange to determine the threshold to separate live- and dead- cells and staining efficiency. The heat-killed and live cells are mixed in different percentages followed by staining with SYTOX Orange and analysis by flow cytometry. The percentage cells in live and dead population calculated gate reflected the starting percentages of heat-killed and live cells in premixed populations with correlation coefficient (r) of 0.99. Mean ± SD of at least two biological replicates is plotted. (D) Gating strategy when SYTO 9 and SYTOX Orange were used in combination with Vybrant DiD in order to differentiate cells from debris and, dead cells.

## Slide 5
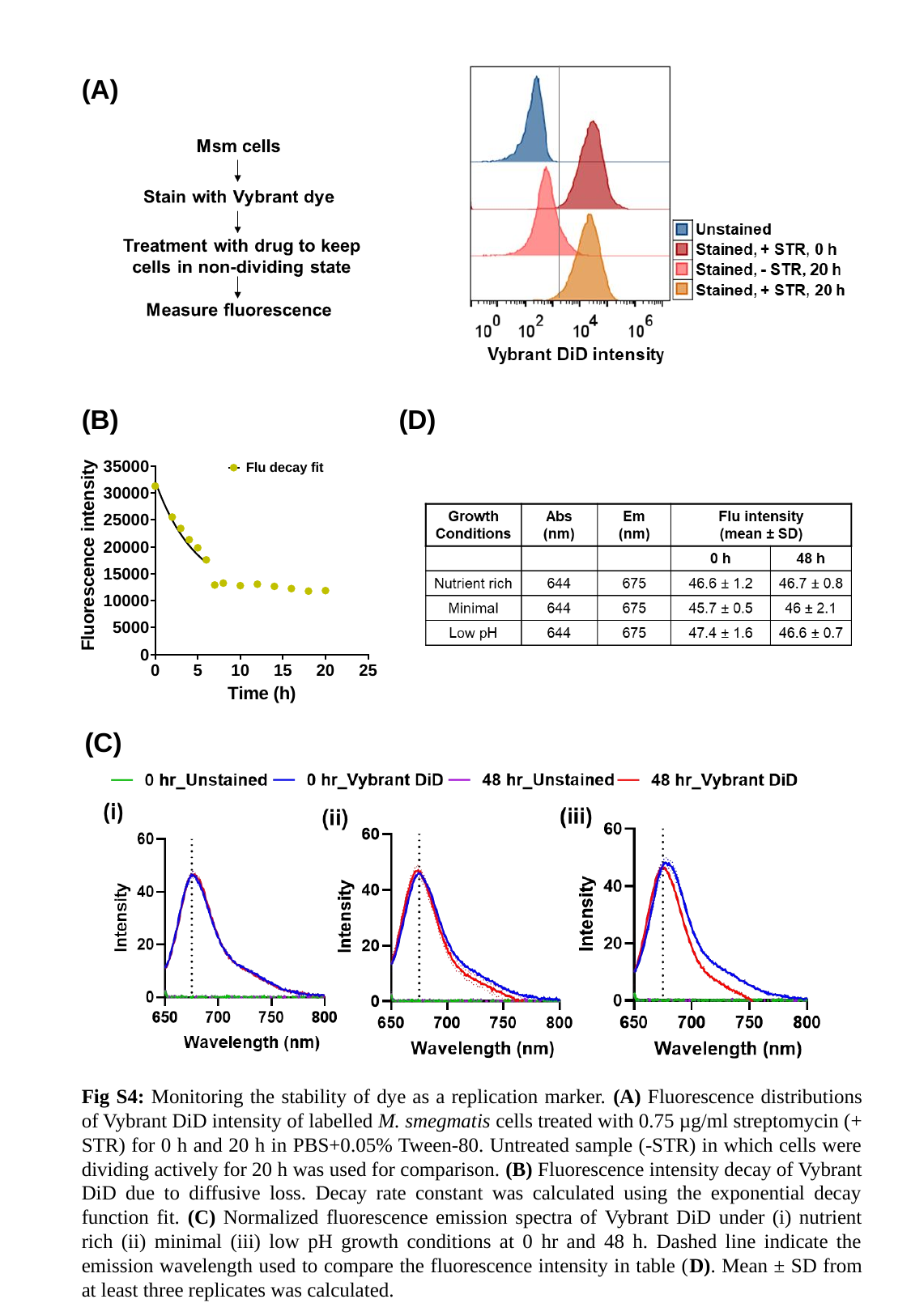

(A)
(D)
(B)
(C)
Fig S4: Monitoring the stability of dye as a replication marker. (A) Fluorescence distributions of Vybrant DiD intensity of labelled M. smegmatis cells treated with 0.75 µg/ml streptomycin (+ STR) for 0 h and 20 h in PBS+0.05% Tween-80. Untreated sample (-STR) in which cells were dividing actively for 20 h was used for comparison. (B) Fluorescence intensity decay of Vybrant DiD due to diffusive loss. Decay rate constant was calculated using the exponential decay function fit. (C) Normalized fluorescence emission spectra of Vybrant DiD under (i) nutrient rich (ii) minimal (iii) low pH growth conditions at 0 hr and 48 h. Dashed line indicate the emission wavelength used to compare the fluorescence intensity in table (D). Mean ± SD from at least three replicates was calculated.

## Slide 6
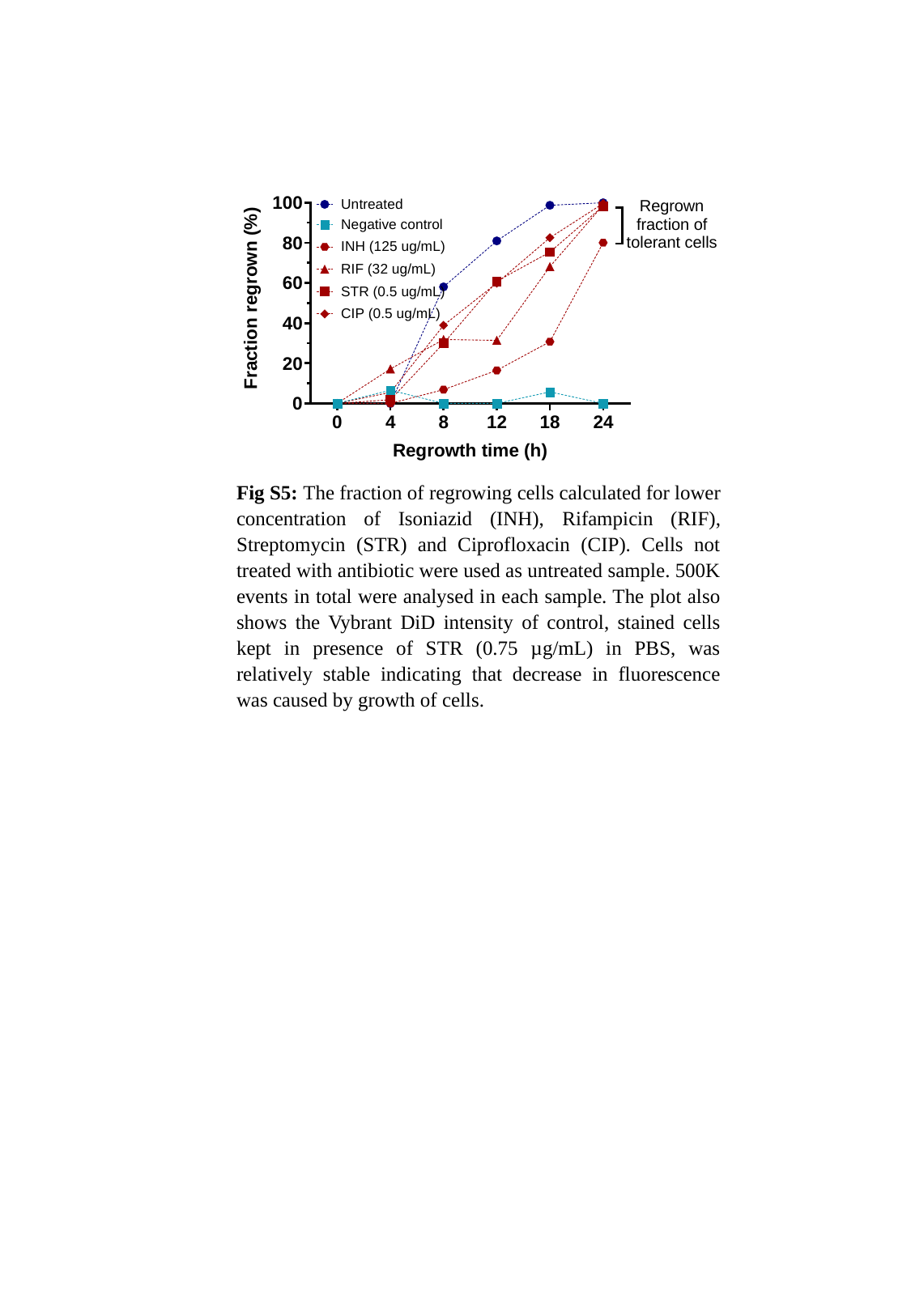

Fig S5: The fraction of regrowing cells calculated for lower concentration of Isoniazid (INH), Rifampicin (RIF), Streptomycin (STR) and Ciprofloxacin (CIP). Cells not treated with antibiotic were used as untreated sample. 500K events in total were analysed in each sample. The plot also shows the Vybrant DiD intensity of control, stained cells kept in presence of STR (0.75 µg/mL) in PBS, was relatively stable indicating that decrease in fluorescence was caused by growth of cells.
