# Supplementary material for: Rapid and efficient labeling by a selective organic fluorophore probe highlights heterogeneity of mycobacterial populations and persister resuscitation
Source: PLoS One. 2025 Dec 18;20(12):e0338563. doi: 10.1371/journal.pone.0338563 (PMC12714215; doi:10.1371/journal.pone.0338563)
Supplement: S2 File — (PDF) [file pone.0338563.s002.pdf]

Minimal data set

Rapid and efficient labeling by a selective organic fluorophore probe highlights heterogeneity of mycobacterial populations and persister resuscitation

Priyanka Chauhan[1], Sander van Otterdijk[1], Susanna Commandeur[1#a], Dirk Bald[1*], Frank J. Bruggeman[1*]

[1]Amsterdam Institute for Life and Environment (A-LIFE), AIMMS, Faculty of Science, Vrije Universiteit Amsterdam, De Boelelaan 1108, 1081 HZ Amsterdam, The Netherlands

[#a] Current affiliation: Department of Bacteriology, Host Pathogen Interaction and Diagnostics Development, Wageningen Bioveterinary Research (WBVR), Wageningen University & Research (WUR), 8221 RA Lelystad, The Netherlands

*Corresponding author

Email: f.j.bruggeman@vu.nl or d.bald@vu.nl

Fig. #	Fold-increase	S.D	# samples
**Fig. 1A**			
5 min			
1.25 μM	37.56	1.5	3
2.5 μM	147.49	2.7	3
5 μM	314.64	1.4	3
10 min			
1.25 μM	36.9459	1.8	3
2.5 μM	117.30	0.2	3
5 μM	357.7	2.1	3
20 min			
1.25 μM	82.4	1.4	3
2.5 μM	165.4	1.7	3
5 μM	398.12	2.6	3

Fig. #	Mean	S.D	Statistical method used	P value	# samples (biological/technical)
**Fig. 1B**					
**Vehicle**	304.5	205.7681	Two-way ANOVA tests for unequal variances with Sidak's multiple comparisons test using selected adjusted *p*-values	***p* < 0.01, ****p* < 0.001, *****p* <0.0001; ns, non-significant	6 (2/3)
Msm	286	181.0193			
Mtb	225.5	103.9447			
Mm	507	0			
Cg	210	77.78175			
Bs	165.5	19.09188			
Pa	165.5	14.84924			
Ec	222	93.3381			
Sa					
Stained					
Msm	30324.5	7913.232			
Mtb	17784.5	491.4392			
Mm	13380.5	2194.152			
Cg	68200	6222.54			
Bs	2521	347.8965			
Pa	2012	1117.229			
Ec	429	74.95332			
Sa	736	100.4092			

Fig. #	Mean	Curve fitting	S.E of the fit	Number of point in fit
**Fig. 2C**				
**Flu intensity data**				
0	60853.9	Nonlinear fit: Exponential growth equation<br><br>Goodness of fit parameters:<br>• Degree of freedom:15<br>• R squared: 0.96	0.0148	17
1	42324.8			
2	29234.3			
3	32721			
4	26082.4			
5	20838			
6	13232.3			
7	6100.13			
8	5261.86			
9	3750.49			
10	3114.66			
12	2403.81			
14	2082.57			
16	1508.38			
18	1168.14			
20	904.606			
22	339.515			
**Cell count data**				
0	223095	Nonlinear fit: Exponential growth equation<br><br>Goodness of fit parameters:<br>• Degree of freedom:14<br>• R squared: 0.96	0.0107	17
1	276553			
2	258918			
3	257122			
4	336428			
5	407683			
6	627575			
7	1028430			
8	1350980			
9	1894360			
10	2540150			
12	2773890			
14	4124060			
16	6203650			
18	7969250			
20	11903904			
22	17064483			

Fig. #	Mean	S.D	Statistical method used	P value	#samples (biological/technical)
**Fig. 2E**					
**OD_{600}(pH 5.8) time points**					
0	0	0	Paired t test (*P<0.01)	0.3102 ns; non-significant	6 (3/2)
4	1.040085	0.056689			
8	1.791565	0.33803			
11	3.084963	0.02061			
14	4.466635	0.13305			
17	5.281121	0.157255			
20	6.325526	0.094457			
**Flu intensity (pH 5.8)**					
0	0	0			
4	1.39	0.014142			
8	2.281058	0.026788			
11	3.049304	0.013157			
14	3.577482	0.01058			
17	4.67059	0.041593			
20	5.194793	0.007365			
**OD_{600}(minimal)**					
0	0	0	Paired t test (*P<0.01)	0.013 ns; non-significant	6 (3/2)
2	0.098536	0.001414			
5	0.196323	0.005201			
7	0.918225	0.011632			
9	1.405287	0.049903			
11	2.017768	0.010985			
13	2.321196	0.029976			
17	2.420732	0.001035			
20	2.781251	0.026516			
**Flu intensity (minimal)**					
0	0	0			
2	0.050886	0.000162			
5	0.191165	0.007376			
7	0.833412	0.110244			
9	1.171458	0.180585			
11	1.461587	0.466582			
13	1.605677	0.54565			
17	1.840361	0.671211			
20	2.154344	0.642602			

All data extracted from images for analysis and all files related to Fig. 3 can be accessed via this link : https://github.com/SanderSMFISH/Vybrant-DiD-staining-of-mycobacterial-cells-analysis/tree/main/Figure3_Macrophage_infection

**Figure 3B**	**Fraction of cells (%)**	
Time points	$DsRed^{+}$ Vybrant DiD^{dim}	$DsRed^{+}$ Vybrant DiD^{+}
0	0	100
12	1.591459	98.40854
24	15.77449	84.22551
36	40.75368	59.24632
48	53.2473	46.7527

**Figure 3C**				
Time points	Mean	SD	N	Curve fitting
0	38970.86	16665.84	256	Nonlinear fit Exponential growth equation
12	20207.59	16176.46	247	
24	18825.01	19256	198	
36	8784.031	8639.09	95	
48	7231.867	8891.535	256	

Fig. #	Mean	S.D	Statistical method used	P value	#samples
**Fig. 4B**					
**Unstained STR (0.5 µg/mL) Time points (days)**			One-way repeated measures ANOVA/Bonferroni : compare selected pairs (*P<0,05)	0.999 ns; non-significant	3
0	1	0			
1	0.056867	0.00383			
2	0.047582	0.000547			
**Stained STR (0.5 µg/mL) Time points (days)**					
0	1	0			
4	0.051451	0.000547			
8	0.023598	0.004924			
**Vehicle STR (0.5 µg/mL) Time points(days)**					
0	1	0			
4	0.038685	0.0004			
8	0.040619	0.002735			
**Unstained STR (0.75 µg/mL) Time points (days)**			One way repeated measures ANOVA/Bonferroni : compare selected pairs (*P<0,05)	>0.999 ns; non-significant	3
0	1	0			
1	0.000156	0.00002			
2	0.000155	0.00004			
**Stained STR (0.75 µg/mL) Time points (days)**					
0	1	0			
1	0.000433	0.00003			
2	0.000847	0.00007			
**Vehicle STR (0.75 µg/mL) Time points(days)**					
0	1	0			
1	0.00013	0.000012			
2	0.000387	0.00002			

**Fig. 4C**					
Expected	Measured				Correlation
	Mean	SD	%SEM (inset)	N	
50	51.5	0.408248	7.97	3	Pearson r = 0.9994<br>R squared= 0.9988<br>P value (two-tailed)= <0.0001
25	24.5	2.857738	5.79	3	
10	8.865	2.082795	4.74	3	
5	4.9725	0.209026	2.1	3	
1	1.2425	0.117863	4.74	3	
0.5	0.4425	0.051235	5.79	3	
0.1	0.0975	0.015546	0.4	3	

**Fig. 4E**								
	Bio. Rep.							Statistical method used
	1	2	3	Mean	SD	SEM	N	Unpaired two-tailed t test<br>P value (two-tailed)= 0.001)
Exponential	0.039	0.038	0.052	0.043	0.08	0.005	3	
SHX treated	0.29	0.3	0.4	0.33	0.061	0.035	3	

**Fig. 5 C**	**Fraction regrown (%)**					
Time (h)	INH (250 ug/mL)	RIF (64 ug/mL)	STR (0.75 ug/mL)	CIP (1 ug/mL)	Untreated	N
0	0	0	0	0	0	500K events analyzed per sample in flow cytometer
4	2.06224	0	0.359075	4.801425	0	
8	11.58458	7.533034	17.84737	11.4567	58.01583	
12	18.7752	7.475053	60.27132	27.593	80.92599	
18	43.39579	75.15182	68.46414	36.38699	98.66249	
24	75.70895	95.23177	97.86564	86.83474	99.89902	

Fig. 6D			
Untreated			
Days of hypoxia	Mean	SD	N
0	6.046973362	0.09471	3
1	7.676091	0.032355	3
2	7.743923	0.185458	3
3	7.6393768	0.084278	3
7	7.531570855	0.185746	3
CIP (5 µg/mL)			
	Mean	SD	N
0	0	0	3
1	7.813555	0.0869832	3
2	7.753253	0.03521213	3
3	7.088984	0.04000347	3
7	6.753253	0.03521213	3
INH (350 µg/mL)			
	Mean	SD	N
0	3.954242	0.02	3
1	7.880211	0.03235543	3
2	7.333142	0.2982945	3
3	7.228865	0.07055275	3
7	6.469759	0.01041092	3
MTNZ (120 µg/mL)			
	Mean	SD	N
0	8.307607	0.2598689	3
1	7.747077	0.09310625	3
2	7.215682	0.1207005	3
3	5.40224	0.00608124	3
7	1.676091	0.03235543	3

All data analysis files related to Fig. 6F can be accessed via this link :

https://github.com/SanderSMFISH/Vybrant-DiD-staining-of-mycobacterial-cells-analysis/tree/main/Figure6E_Hypoxia_resurcitation
